# AlgaeOrtho, a bioinformatics tool for processing ortholog inference results in algae

**DOI:** 10.3389/fmicb.2025.1541898

**Published:** 2025-03-04

**Authors:** Mary-Francis LaPorte, Neha Arora, Struan Clark, Ambarish Nag

**Affiliations:** ^1^Department of Plant Sciences, University of California, Davis, Davis, CA, United States; ^2^Department of Biology, Skidmore College, Saratoga Springs, NY, United States; ^3^Computational Science Center, National Renewable Energy Laboratory, Golden, CO, United States

**Keywords:** bioengineering, algae, metabolic engineering, bioinformatics, nutraceuticals, protein orthology

## Abstract

**Introduction:**

Microalgae constitute a prominent feedstock for producing biofuels and biochemicals by virtue of their prolific reproduction, high bioproduct accumulation, and the ability to grow in brackish and saline water. However, naturally occurring wild type algal strains are rarely optimal for industrial use; therefore, bioengineering of algae is necessary to generate superior performing strains that can address production challenges in industrial settings, particularly the bioenergy and bioproduct sectors. One of the crucial steps in this process is deciding on a bioengineering target: namely, which gene/protein to differentially express. These targets are often orthologs which are defined as genes/proteins originating from a common ancestor in divergent species. Although bioinformatics tools for the identification of protein orthologs already exist, processing the output from such tools is nontrivial, especially for a researcher with little or no bioinformatics experience.

**Methods:**

The present study introduces AlgaeOrtho, a user-friendly tool that builds upon the SonicParanoid orthology inference tool (based on an algorithm that identifies potential protein orthologs based on amino acid sequences) and the PhycoCosm database from JGI (Joint Genome Institute) to help researchers identify orthologs of their proteins of interest in multiple diverse algal species.

**Results:**

The output of this application includes a table of the putative orthologs of their protein of interest, a heatmap showing sequence similarity (%), and an unrooted tree of the putative protein orthologs. Notably, the tool would be instrumental in identifying novel bioengineering targets in different algal strains, including targets in not-fully annotated algal species, since it does not depend on existing protein annotations. We tested AlgaeOrtho using three case studies, for which orthologs of proteins relevant to bioengineering targets, were identified from diverse algal species, demonstrating its ease of use and utility for bioengineering researchers.

**Discussion:**

This tool is unique in the protein ortholog identification space as it can visualize putative orthologs, as desired by the user, across several algal species.

## 1 Introduction

Microalgae are a prominent feedstock for biofuels and other industrially important bioproducts because of their prolific reproduction, high biomolecule (such as lipid, protein, carbohydrate, and pigments) accumulation, and aquatic nature that does not compete for arable land (Kumar et al., 2020). Ideally, an industrial algal strain should possess high abiotic and biotic stress tolerance, enhanced bioproduct yield, and robustness to outdoor cultivation (Griffiths and Harrison, 2009; Arora et al., 2018; Chen et al., 2018; Mathimani and Mallick, 2018). However, no wild type strain is ideal for industrial use, even the most advantageous microalgal strain(s) have natural physiological limitations (Araújo et al., 2021; Verdelho Vieira et al., 2022). Bioengineering desirable transgenic algae strains is a way to improve the natural capabilities of algal species to address industry needs, for boosting the productivity of value-added products such as nutraceuticals, and cosmeceuticals (Banerjee et al., 2016; Kumar et al., 2020). Down-selecting natural physiological advantages from diverse algal strains can aid in the identification of metabolic engineering targets, however the breadth of microalgae diversity has not yet been explored for its relevance to biofuels and nutraceuticals (Araújo et al., 2021; Verdelho Vieira et al., 2022). To bioengineer an algal strain, potential target(s) can be identified through comparative omics (including genomics, proteomics and transcriptomics) wherein selection of a gene/protein empirically shown to be associated with a phenotype of interest such as high growth, salinity tolerance, or enhanced industrially-beneficial bioproduct synthesis in one species is used as a template to find similar gene/proteins in other target algae (Kumar et al., 2020). The identified genes/proteins can be orthologs, which are defined as genes/proteins originating from a common ancestor and conserved in divergent species. These protein orthologs can be identified using publicly available genomic resources, including the US Department of Energy (DOE) funded PhycoCosm from JGI, which hosts and maintains multi-omics data and tools for researchers (Grigoriev et al., 2012, 2021; Nordberg et al., 2014).

Indeed, several algorithms and tools including OrthoFinder, OMA2011, inParanoid, and SonicParanoid have been developed to identify protein orthologs between groups of species (Li et al., 2003; Ostlund et al., 2010; Altenhoff et al., 2011; Emms and Kelly, 2015, 2019). Among the above listed tools, SonicParanoid is a fast and accurate command line tool for identifying protein orthologs across multiple species (Cosentino and Iwasaki, 2019). SonicParanoid identifies groups of orthologous proteins between the proteomes of a set of species of interest. With bioinformatic processing of the results from SonicParanoid, researchers can identify orthologs of specific proteins of interest across species to search for novel metabolic engineering targets *in silico*. However, processing the output of SonicParanoid requires bioinformatics experience, which may impede researchers who are focused on quickly identifying protein targets of interest.

**Figure 1:**
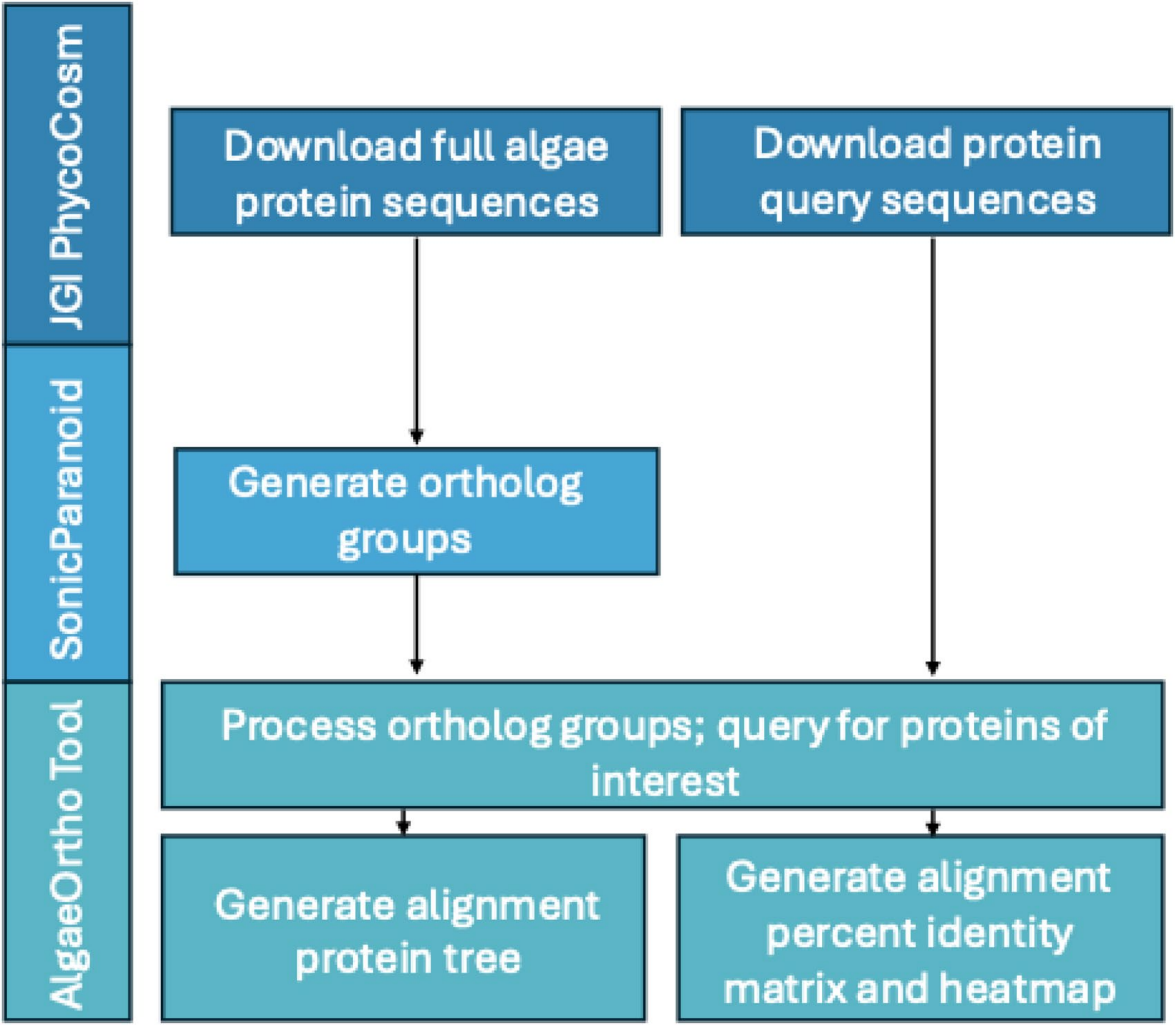
The full workflow for the AlgaeOrtho tool to search for the query proteins with outputs composed of an alignment protein tree and a heatmap of the percent identity matrix for the putative orthologs.

**Figure 2:**
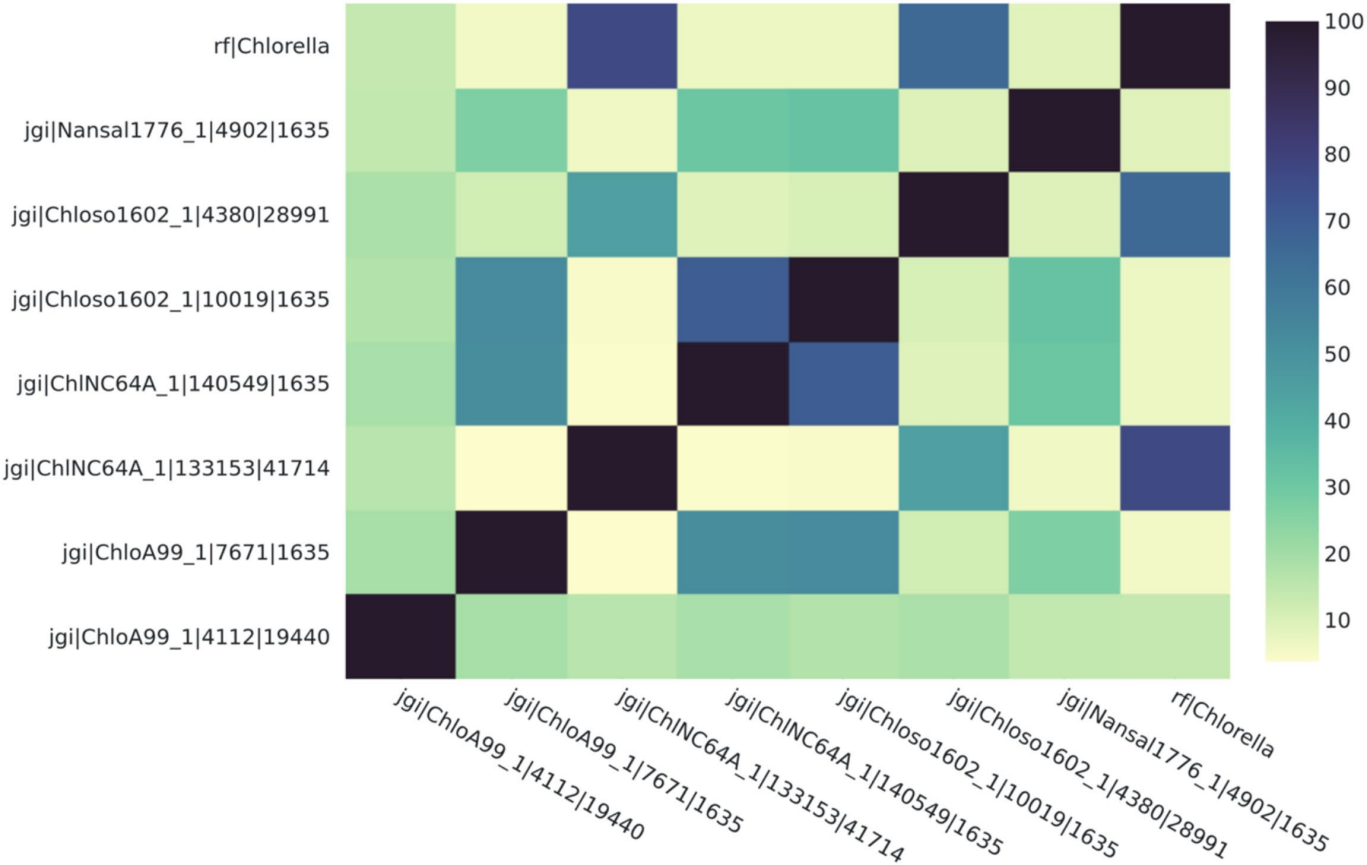
*Chlorella* HS2 bZIP1 (above, labeled “Chlorella”) has high sequence similarity with *Chlorella* var*iabilis NC64A* (“ChlNC64A”) and *Chlorella sorokiniana* UTEX 1602 (“Chloso1602”) orthologs (77.1 and 66.1% respectively). The heatmap depicts Percent Identity Matrix (PIM) where the values are percent sequence similarity (%) between putative orthologs. The names on each axis reflect the species from which the ortholog sequence was identified. The naming convention of the labels reflects the JGI naming convention of proteins from proteome sequences: <jgi>, which denotes a sequence origin of JGI | <species identification code>, originating from the JGI system | <protein identification number>, which is species and JGI specific| <ortholog group number>, which was generated by SonicParanoid.

**Figure 3:**
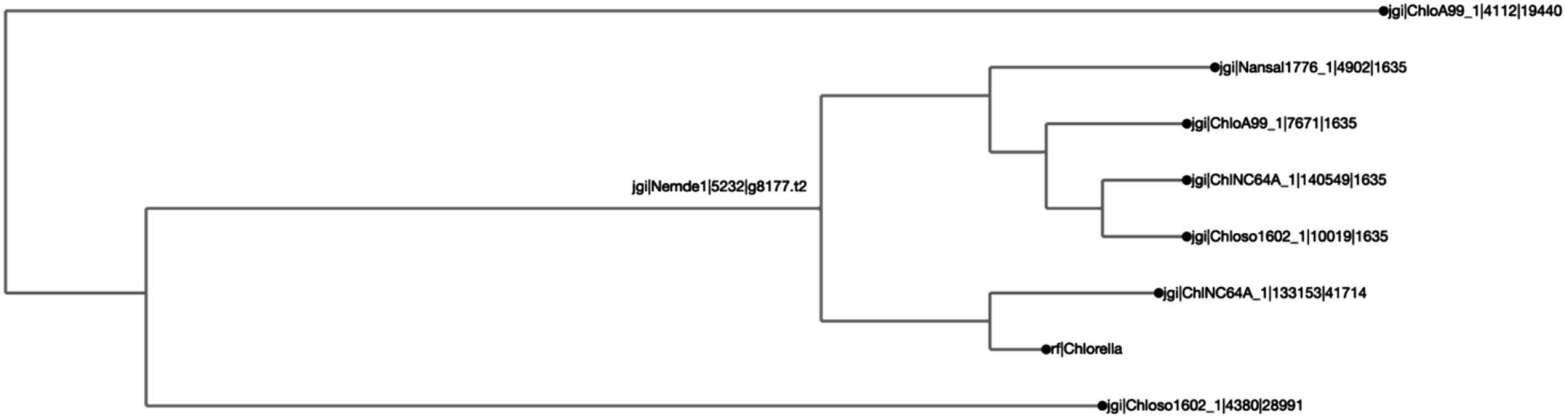
*Chlorella* HS2 bZIP1 is clustered with one *Chlorella variabilis* NC64A (“ChlNC64A”) ortholog, and shares a common ancestor with two other orthologs, one from *Chlorella sorokiniana* UTEX 1602 (“Chloso1602”) and another from *Chlorella* sp. A99 (“ChloA99”). The clustering was calculated by Clustal Omega, and the distance calculated by BioPython’s ‘Phylo Tree Construction’ tools and entries are rooted to the mean. The length of the line reflects phylogenetic distance of the sequences. The names on each axis reflect the species from which the ortholog sequence was identified. The naming convention of the labels reflects the JGI naming convention of proteins from proteome sequences: <jgi>, which denotes a sequence origin of JGI | <species identification code>, originating from the JGI system | <protein identification number>, which is species and JGI specific| <ortholog group number>, which was generated by SonicParanoid.

In this study, we introduce the AlgaeOrtho tool, an application to process SonicParanoid ortholog groups to help identify novel regions of interest *in silico* for bioengineering experiments. The AlgaeOrtho tool can be used to identify orthologs of the protein of interest in diverse algal strains, even for species that have yet to be annotated, which can then be tested as potential targets for bioengineering across diverse classes of algae (Figure 1). The tool generates a heatmap of the sequence similarities and an unrooted protein tree showing the clustering of the identified putative orthologs (Supplementary Figure S1; Figures 2, 3). Notably, the AlgaeOrtho tool includes an accessible and easy-to-use application interface that allows the user to upload their protein(s) of interest and visualize the ortholog relatedness. To best of our knowledge, this tool is unique in the protein ortholog identification space to visualize putative orthologs across several algal species as per the user preference. It is built on established ancillary resources, including SonicParanoid and DOE’s PhycoCosm database. Other existing tools execute similar ortholog analysis tasks, but none fits the niche served by AlgaeOrtho. For instance, PhycoCosm’s comparative genomics tools compare specific, pre-loaded pairs of species, while AlgaeOrtho can be used for any algal species specified by the user. As another example, the OrthoFinder tool compares two or more entire genomes to one another (Emms and Kelly, 2015, 2019). In contrast, the AlgaeOrtho tool is helpful for simultaneously identifying specific proteins orthologous to one another in several algal species. Moreover, Basic Local Alignment Search Tool (BLAST) and related resources are unable to perform multiple sequence alignment (Altschul et al., 1990) while the multi-sequence alignment tool Clustal Omega (which is utilized in AlgaeOrtho) does not visualize multiple orthologs (Sievers et al., 2011). Therefore, we introduce the AlgaeOrtho tool to aid the identification of protein orthologs across many algal species, leveraging publicly available sequences and software, with the goal of identifying new bioengineering targets *in silico*.

## 2 Methods

### 2.1 Collection of protein sequences

The protein sequences used in this study were downloaded in bulk (in FASTA format) for all algal species from the JGI PhycoCosm website (https://phycocosm.jgi.doe.gov/phycocosm/home). The initial input for this software is a directory that contains all sequences that were used as the bases for the orthologous groups. The directory housed 92 files containing the full protein sequences for each of these species (Supplementary Table S1). In total, 1,122,751 sequences were included in the tool. More detailed instructions on how to download bulk sequences from this web portal can be found in the Supplementary Section S1. The AlgaeOrtho tool can be used to identify putative orthologs to proteins of interest for any algal species for which the user has sequences data (or can access sequence data through JGI).

### 2.2 Case study queries

To showcase this software in action, this work presents case studies focusing on three different proteins of interest, (1) basic region/leucine zipper motif 1 (bZIP1) transcription factor from *Chlorella* sp. HS2, found in all species of genus *Chlorella*, (2) lycopene β-cyclase (LCYB) as found across all the species in the taxa Ochrophyta (Wichuk et al., 2014; Kim et al., 2019; Ren et al., 2021) and (3) cellulose synthase (*CesA*) that has been identified to alter cell wall composition in a *Nannochloropsis* species, through targeted modification using CRISPR-Cas9 (Jeong et al., 2020). The bZIP1 and LCYB proteins were selected because of their relevance to stress responses in algae and land plants; a desirable phenotype in the context of bioengineering algae for enhancing biofuel and bioproduct yield in outdoor cultivation systems (Zhu et al., 2018; Choi et al., 2022). On the other hand, the case study for CesA demonstrates the utility of the AlgaeOrtho for proteins related to cell wall biosynthesis, a relatively complex biochemical network with many related proteins, to ease the downstream processing and extraction of metabolites from algal biomass (Jeong et al., 2020).

The first target protein belongs to the class of bZIP transcription factors that have been associated with universal stress responses, relating to withstanding oxidative, salt, and/or drought stressors in both algae and land plants; a desirable trait for an alga to be cultivated outdoors (Zhu et al., 2018; Choi et al., 2022). In particular, our study focuses on the bZIP1 identified from *Chlorella* sp. HS2, since its overexpression resulted in higher lipid yields in the transgenic strain cultivated in heterotrophic media (Kim et al., 2019). The authors reported that this transcription factor exhibited the highest homology to *Nannochloropsis salina* bZIP1 as compared to any of the bZIPs identified and tested (Kim et al., 2019; Lee et al., 2020). Since targeting this protein and its homolog was successful for bioengineering in two disparate algal species, results from this case study may be relevant for bioengineering in other algae for higher lipid accumulation.

The second target protein, LCYB is an enzyme at a branching point in the carotenoid biosynthesis pathway upstream of both the alpha and beta branches of carotenoid biosynthesis. LCYB has been shown to regulate β-carotene and plays a role in halotolerance (Chen et al., 2011; Liang et al., 2017). This enzyme has been associated with stress tolerance in both land plants and algae and has been thoroughly studied as a bioengineering target (Chen et al., 2011; Liang et al., 2017; Zhao et al., 2020). In addition, since LCYB is associated with carotenoid accumulation, it is relevant to engineering of algal strains specifically to produce β-carotene, which is already a major algae-based nutraceutical product (Arriola et al., 2018; Verdelho Vieira et al., 2022).

*CesA1*, a mutation created to disrupt the cell wall synthesis in *N. salina* resulting in thinner cell walls with lower cellulose content (Jeong et al., 2020). Thinner cell walls in algae are particularly advantageous for biofuel production in the context of rendering the downstream processes including digestibility, harvesting and extraction more energy efficient (Donk et al., 1997; Schwede et al., 2013). The authors introduced the *CesA1* mutation using CRISPR-Cas9 to reduce the cell wall thickness to aid the lipid extraction, thereby identifying this mutation as a “potential target for developing microalgae-based biofuel production” (2020). Notably, other biofuel-relevant algae such as *Nannochloropsis* or *Chlorella* species could be relevant targets for future bioengineering efforts by identifying putative orthologs using AlgaeOrtho.

### 2.3 Ortholog inference

Ortholog groups were inferred using SonicParanoid (Cosentino and Iwasaki, 2019). To summarize the tool in brief: SonicParanoid is a graph-based algorithm, which for *N* input protein sequences, conducts protein alignment for all *N*×(*N*-1) sequences, and all pairwise sequence alignments thereof (Cosentino and Iwasaki, 2019). This method can robustly align a larger number of sequences more quickly than similar graph-based methods such as InParanoid since it omits unnecessary bootstrapping steps (Remm et al., 2001; Cosentino and Iwasaki, 2019). Among its outputs, SonicParanoid generates a list of each of the protein ortholog groups. Each row of this list is a putative protein ortholog group, containing the names and accession numbers for each protein found in that group, including protein orthologs from different species. Our software pipeline processes the results using the Python Pandas library (The Pandas Development Team, 2024) to reformat the results from a non-standard format into a tabular data frame (which can be downloaded by the user for further use) and easily queried for a particular protein of interest. Instructions for using SonicParanoid are detailed in the Supplementary Section S2.

### 2.4 Querying and reporting putative orthologs

A query file, which includes the protein sequence(s) in FASTA format for which the user is looking for putative orthologs, is the starting point for this pipeline. The orthologs identified by the pipeline as related to the protein(s) of interest are then aligned with each other using Clustal Omega (Sievers et al., 2011). Clustal Omega is also used to calculate a matrix of the percent identity alignments between the sequences that the user can visualize using a matrix heatmap (generated using Python’s Plotly Express library). Thus, the initial alignment is conducted through SonicParanoid to create the ortholog groups which are subsequently aligned through Clustal Omega to be included in the percent identity matrix and the resulting protein tree. At this step, the query file can include any number of protein sequences of interest (see Supplementary material). Once aligned, an orthologous group of protein sequences is converted into a Newick file using the BioPython library’s tree tool called “Bio.Phylo.TreeConstruction,” for visualization of alignments between the orthologs in our software tool, or for the users to download to use in their visualization software of choice. This tree tool calculates a distance matrix between sequences and uses this distance matrix to construct a distance tree using the neighbor joining method. The tree is rooted to the midpoint, as a default, using the root_to_midpoint() function from Biopython’s “Bio.Phylo” library. The midpoint is calculated from the two most distant taxa in the tree. The user can unselect this option to see an un-rooted tree. Instructions for the setup and use of the AlgaeOrtho application are described in the Supplementary Section S3.

### 2.5 Availability of data and materials

The datasets supporting the conclusions of this article are available in the Joint Genome Institute’s PhycoCosm repository, https://phycocosm.jgi.doe.gov/phycocosm/home. The sequence file names searchable on the database are detailed in Supplementary Table S1.

## 3 Results

**Figure 4:**
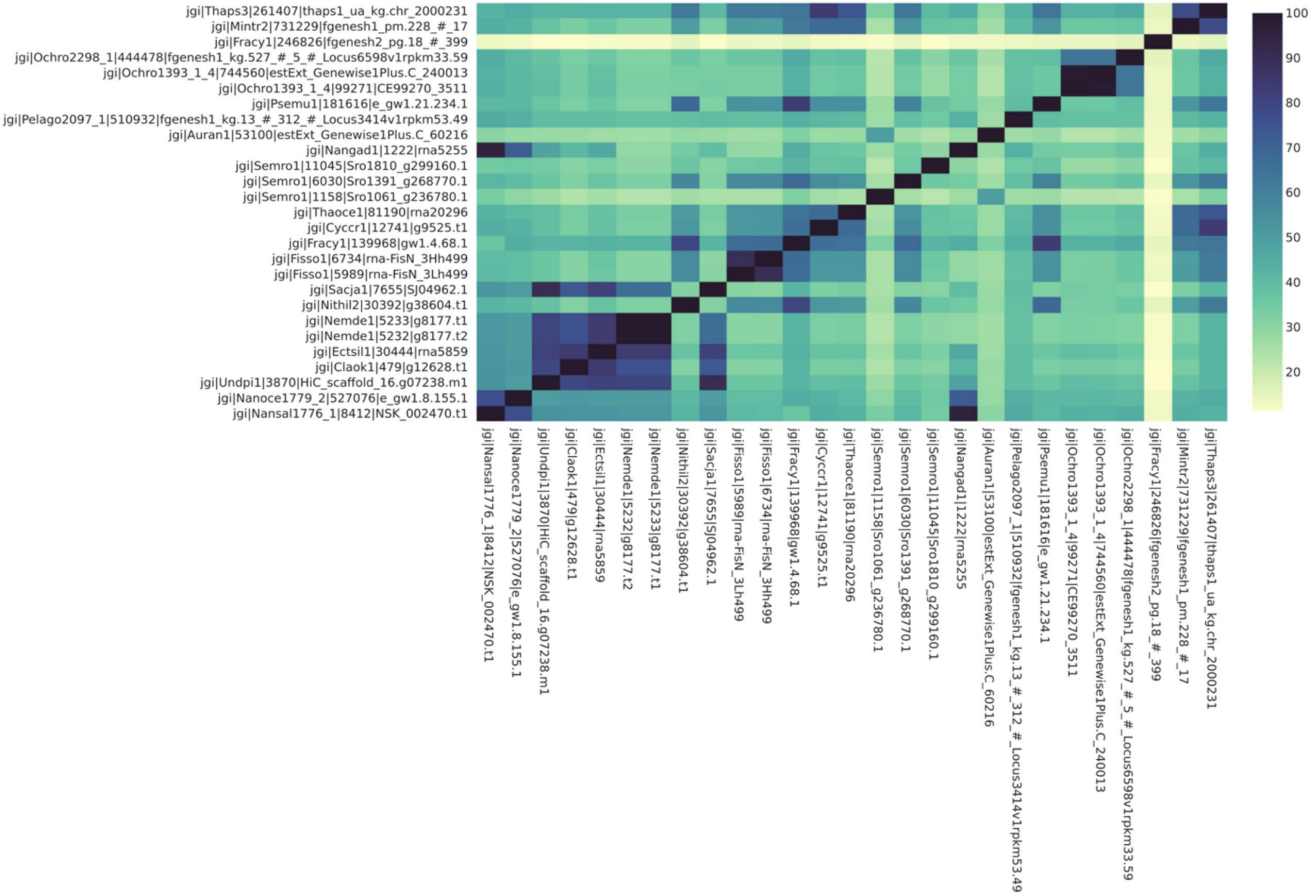
Some proteins identified as LCYB in the Ochrophyta have high protein sequence identity similarity (>70%) with protein orthologs found in other species. Notably, the economically important species *Ectocarpus siliculosus* (Ectsil1) has high similarity with other human-edible species for this protein, which is known to be related to color, taste, and therefore consumer preference. The names on each axis reflect the species from which the ortholog sequence was identified. The naming convention of the labels reflects the JGI naming convention of proteins from proteome sequences: <jgi> | <species identification code>| <ortholog group number>| <protein identification code>. This signifies: The origin in the JGI database | a code specific to the JGI system | ortholog number generated by SonicParanoid | a protein code specified by JGI.

**Figure 5:**
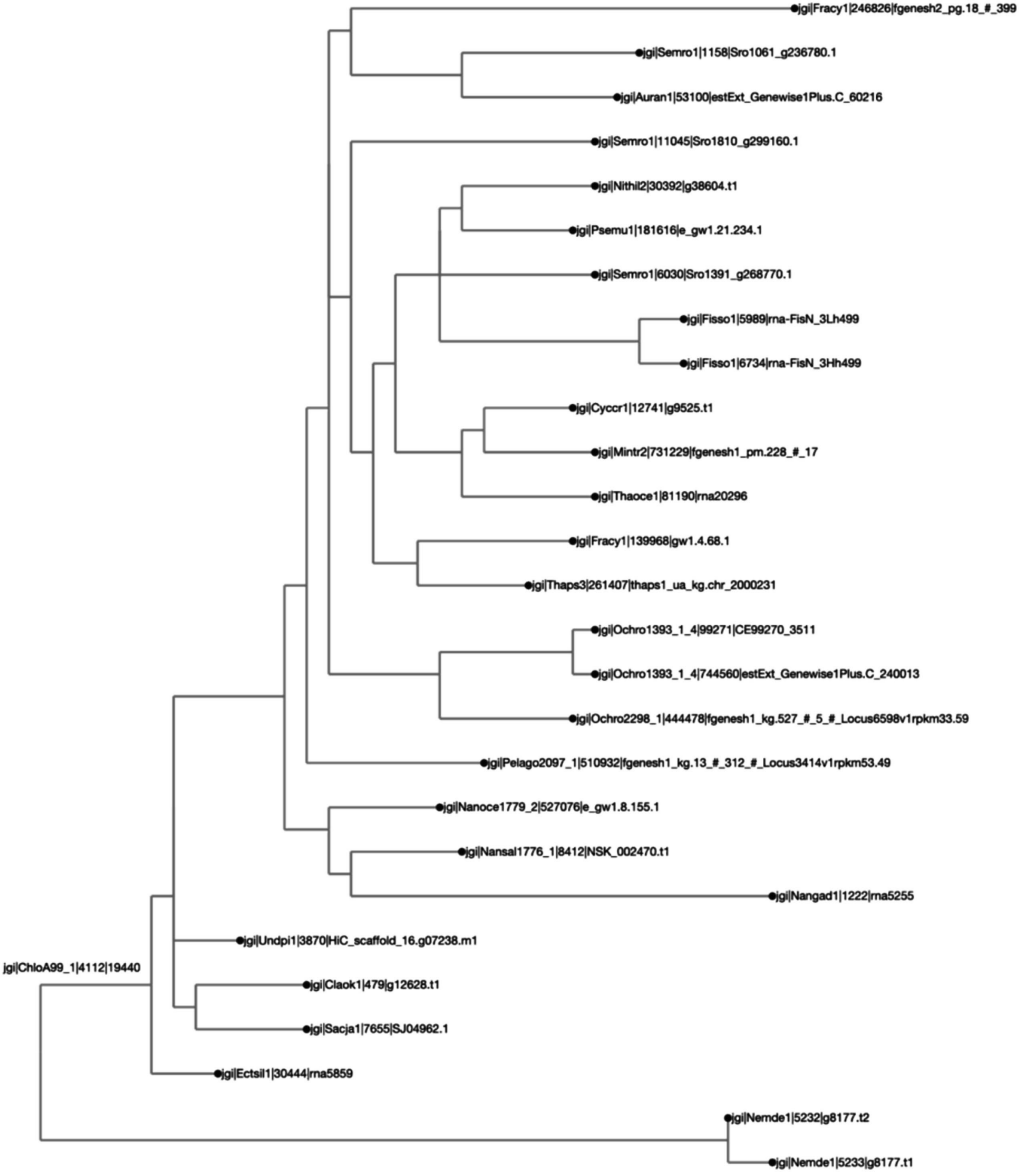
Different protein orthologs originating from the same genus or species tend to belong to the same clade, as expected. The clustering was calculated by Clustal Omega, and the distance calculated by BioPython’s “Phylo Tree Construction” tools and entries are rooted to the mean. The names on each axis reflect the species from which the ortholog sequence was identified. The naming convention of the labels reflects the JGI naming convention of proteins from proteome sequences: <jgi> | <species identification code>| <ortholog group number> | <protein identification code>. This signifies: The origin in the JGI database | a code specific to the JGI system | ortholog number generated by SonicParanoid | a protein code specified by JGI.

To test the efficacy of this tool, three case studies were conducted to determine protein orthologs of three bioengineering-relevant targets, a bZIP (Figures 2, 3), a LCYB protein (Figures 4, 5), and CesA1 (Supplementary Figures S5, S6). The ortholog groups were generated using SonicParanoid, and the results were processed using the AlgaeOrtho tool, which filtered out the putative orthologs associated with the proteins of interest, aligned them using Clustal Omega, and calculated a percent identity matrix (sequence similarity metric), visualized the percent identity matrix as a heatmap (Figures 2, 4) and the alignment as an unrooted protein tree (Figures 3, 5).

We found that putative bZIP orthologs in *Chlorella* species had sequence similarities ranging from 3.8 to 77.1% among 92 algal species (Figure 2). The percent identity matrix revealed that *Chlorella* HS2 sp. version of bZIP1 (Kim et al., 2019) (Figure label: “Chlorella”) has the highest similarity (77.1%) with the putative ortholog from *Chlorella* var*iabilis* NC64A (“ChlNC64A”) (Blanc et al., 2010). The next highest sequence (70.0%) similarity of *Chlorella* sp. HS2 bZIP1 was recorded for *Chlorella sorokiniana* UTEX 1602 (“Chloso1602”) (Arriola et al., 2018). The relative clustering of orthologs found in the first case study was the same for both the protein tree and percent sequence identity methods (Figure 3). *Chlorella* HS2 bZIP1 was placed in the same clade as one bZIP from *C. variabilis* NC64A (closest homolog), followed by two others from *C. sorokiniana* UTEX 1602 (JGI species identification code: 4380) and *Chlorella* sp. A99 (JGI species identification code: 4112), an algae-animal symbiont (Hamada et al., 2018). Notably, the *Chlorella* HS2 bZIP1 did not cluster most closely with the *Nannochloropsis* species.

For the second case study, we looked at the orthologs of LCYB across all Ochrophyta. The query file that was downloaded from PhycoCosm included all annotated bZIPs from Ochrophyta. Lycopene β-cyclase (LCYB) orthologs in the Ochrophyta had percent sequence similarity values ranging from 11.5 to 98.5% (Figure 4). They included proteins that were not in the initial query, and therefore were not previously annotated as LCYB orthologs in PhycoCosm. The ortholog tree representation of the results was consistent with the phylogenetic expectations of the Ochrophyta (Figure 5). Sequences from the same species clustered together, for example in the case of Fisso (*Fistulifera solaris*) (Tanaka et al., 2015), Nemde1 (*Nemacystus decipiens*) (Nishitsuji et al., 2019), Ochro1393 (*Ochromonas* CCMP1393) (Ochromonas sp. CCMP1393 v1.4, 2018), and the *Nannochloropsis* species Nansal (*Nannochloropsis salina*) (Ohan et al., 2019), Nanoce (*Nannochloropsis oceanica*) (Nannochloropsis oceanica CCMP1779 v2.0, 2018), and Nangad (*Nannochloropsis gaditana*) (Corteggiani Carpinelli et al., 2014; Schwartz et al., 2018) (Figure 5).

For the third, a more complex case study, all cellulose synthase CESA-annotated proteins in the Eustigmatophyta were queried. The Eustigmatophyta are members of the Ochrophyta that usually have a cell wall and are found in fresh water (Amaral et al., 2020; Borowitzka, 2018). This clade was chosen because it contains the *Nannochloropsis* genus, particularly *N. saliana*, which was the species in which the CRISPR target was empirically validated for CesA (Jeong et al., 2020). The search resulted in 26 different CESA-annotated proteins from 18 different species accessions from PhycoCosm (Supplementary Table S2). Using the AlgaeOrtho application, a total of 109 putative orthologs were identified in ~70 unique species/strains. 108 of the 109 putative orthologs identified had a 100% sequence similarity with the query file containing sequences from Eustigmatophyta (Supplementary Table S2).

## 4 Discussion

The AlgaeOrtho tool visualizes protein orthologs identified between several algal species using SonicParanoid. This feature can be helpful for identifying bioengineering targets *in silico* between evolutionarily distant species, and for proteins that have not yet been annotated for function. The goal of the bioengineering is to differentially express (over-express, knock-out, or down-regulate) a gene of interest in a species of choice to obtain the desired genotypic outcome. A common method to achieve this is to use the BLAST algorithm to find sequence similarity of the gene of interest to sequences in the genomes of other species. One benefit of tools which focus on proteins rather than gene regions is to identify orthologs with genetic sequences which are not highly conserved. Some sequences might not appear identical at the gene level but encode proteins that perform the same function. This is because multiple codons can encode for the same or similar amino acids so that multiple genetic sequences can end up producing functionally equivalent proteins.

The AlgaeOrtho tool allows for the user to look at several different species simultaneously, as the ortholog groups are produced for the algal species the user selects, including protein sequences from non-publicly available or non-annotated proteomes. Since, over half of algal proteins do not have an annotated function, an annotation-agnostic comparative approach (which does not rely on manual description of protein function) enables the identification of relevant bioengineering target regions in un-sequenced and novel algal species (Blaby-Haas and Merchant, 2019). An example of this was the identification of a putative ortholog within the pennate diatom Fisso (Tanaka et al., 2015). This diatom has been identified for its high levels of triacylglycerol production but is unlikely to be included in a study by virtue of being a non-model organism. However, with AlgaeOrtho, it is possible to screen many species simultaneously for putative orthologs, facilitating the inclusion of a putative ortholog from a diverged, potentially unexpected, species. The ability to look through as many species as desired at once is beneficial for exploratory analyses that would be too difficult to undertake, one by one, using BLAST. This is a benefit of multi-sequence alignment algorithms like SonicParanoid, versus single-sequence alignment algorithms such as BLAST.

The ability to identify putative orthologs of a protein species of interest showcases one of the key advantages of the AlgaeOrtho tool which can include protein sequences in a multi-sequence alignment even if the entire proteomic data is not available, in addition to identifying alternative species with publicly available omics resources in which bioengineering of a similar target could be conducted. This was demonstrated in the first case study, where the *Chlorella* HS2 sp. version of bZIP1 had the highest similarity with a putative ortholog from *C.* var*iabilis* NC64A (“ChlNC64A”), which is a model species for algal-viral interactions (Remm et al., 2001), and the next highest with *C. sorokiniana* UTEX 1602 (“Chloso1602”), which is a feedstock species for lipids (Arriola et al., 2018). Previous studies reported that overexpression of a *bZIP* gene from *Chlorella* sp. HS2 (for which a full genome sequence is not yet publicly available to the best of our knowledge) and its ortholog in *N. salina*, produce an over 70% increase in lipid yield in heterotrophic media (Kim et al., 2019; Lee et al., 2020). Notably, *Nannochloropsis*-contained bZIP1 that was identified as an ortholog of *Chlorella* sp. HS2 bZIP1 did not show a high sequence similarity with the others (<33%), despite being an empirically confirmed ortholog (Lee et al., 2020). This is logical since the *Chlorella* sp. HS2 is more closely related to the other *Chlorella* species than to the *Nannochloropsis* species and would likely have had less evolutionary divergence. It is worth mentioning that although, *Chlorella* HS2 bZIP1 protein sequence has been reported in the literature (Lee et al., 2020), the full proteome of this species is not currently publicly available.

Most importantly, these species may be relevant targets for bioengineering, to test if overexpressing the related gene in the identified species (*C. variabilis* NC64A, *C. sorokiniana* UTEX 1602 and *Chlorella* sp. A99) also results in enhanced lipid content similar to that reported in *Chlorella* sp. HS2 and *N. salina*. Both species have been explored as potentially relevant for biofuels and nutraceuticals, so these sorts of studies could help bioengineer the best traits from one species into the other (Blanc et al., 2010; Du et al., 2018; Kwon et al., 2018). Indeed, bZIPs in plants and algae that have been implicated in halotolerance mechanisms would constitute other possible targets for bioengineering. These bZIPs could be included in the input files and their orthologs could be identified by AlgaeOrtho from a range of algal species. This would be a good next experiment for a bioengineer looking for putative targets across algal species.

The second case study shows that this analytical pipeline was able to identify putative orthologs that have not been identified, since Ochrophyta sequences with high similarity were included, even when not annotated as LCYBs (Figures 4, 5). This is logical, since not all proteins are annotated in all species even in resources like JGI that have robust annotations. There were clusters of species with very high sequence similarity, notably between *Nemacystus decipiens* species (“Nemde1”) (Nishitsuji et al., 2019), *Cladosiphon okamuranus* S strain (“Claok1”) (Nishitsuji et al., 2016), and *Undaria pinnatifida* (“Undpi1”) (Shan et al., 2020) which are brown algae grown for human consumption and nutraceuticals, and *Ectocarpus siliculosus* (“Ectsil”) (Cock et al., 2010), which is a filamentous brown alga. Phylogenetic analysis for LCYB showed that proteins from closely related species tend to have proteins with more-closely aligned sequences. For example, a putative ortholog from a diverged diatom (*Fragilariopsis cylindrus*; “Fracy”) was identified from the analysis. This sequence had the lowest sequence similarities (ranging from 11.3 to 15.8%) with all other included species (Figure 4). To determine if this protein is a true ortholog, further follow-up analyses would be warranted to investigate this protein in relation to orthologs from other commonly studied diatoms. In all cases, the results from this study would necessarily need to be verified (through bioengineering) in the algae before claims could be made about the identification of un-annotated LCYB orthologs.

*Chlorella* (a freshwater alga in the Chlorophyta) and *Nannochloropsis* (a marine microalga in the Ochrophyta) are diverged, which is reflected in their lower percentage sequence similarity between their bZIP1 proteins (Shan et al., 2020). But, since these two proteins have been reported to have the same function, it suggests that bZIPs that are more closely clustered may also have the same function (Chen et al., 2018; Lee et al., 2020). That being said, low sequence similarities are still possible between true orthologs, and it is possible to have false positives (sequences with higher similarity that are not actually orthologous) (Rost, 1999; Peterson et al., 2009). Previous studies have reported that low sequence similarities are possible between true orthologs, and protein sequence similarity may not be able determine the function of a protein (Peterson et al., 2009). There is not necessarily a threshold for which amino acid sequence similarity assures orthology, and the practical cut-off for this may depend on the protein of interest. Nevertheless, sequence similarity is still a common way to identify bioengineering targets (Peterson et al., 2009). Thus, although high sequence similarity between two protein sequences could be evidence that two proteins may have the similar functions, not all orthologs have high sequence similarity. Additionally, ortholog groups from SonicParanoid could also include paralogs (the protein underwent a duplication event, and evolved after a speciation event), which tend to have a lower percent sequence similarity (Peterson et al., 2009). More information about how SonicParanoid identifies orthologs and ortholog groups can be found in the paper in which it is described (Cosentino and Iwasaki, 2019).

The identification of 109 putative orthologs for CESA is notable, as almost all of the identified orthologs had identical sequences (Supplementary Table S2; Supplementary Figures S5, S6). The only identified sequence that was not completely identical was annotated as coming from the species “nan” and therefore not included due to annotation errors. Because a CRISPR target has already been identified in *N. salina*, the species identified by AlgaeOrtho could be promising next steps for bioengineering. Related *Nannocloropsis* species, as well as other industry-relevant strains could be the taxa pursued for *in vitro* validation. Species that only have 1 putative ortholog would be easier targets as gene copy number would not be a confounding factor.

As noted from the three different case studies, AlgaeOrtho could be used for several applications such as biofuels, nutraceuticals and other value- added bioproducts. For instance, carotenoids and fatty acids, abundant in *Chlorella* and *Spirulia*, already hold a place in the nutraceutical market (Kim et al., 2019; Ren et al., 2021). AlgaeOrtho could aid bioengineers in identifying targets to bioengineer strains to boost the yields in algae of interest, across strains that are relevant for algal bioengineering. Furthermore, this tool could be used for evolutionary biology research. For example, AlgaeOrtho could be used to identify protein orthologs across species involved in primary cellular processes related to photosynthesis, flagella function could promote fundamental understanding of algal physiology. Additionally, it could be used for evolutionary genomics research, to look at the similarity of proteins involved in regional adaptation (for example, for halotolerance) across species found in a region of interest. A benefit of using the AlgaeOrtho tool is that the user can specify which species are included in their query and augment it with any data that they have access to, according to their research application.

Importantly, to confirm the accuracy of AlgaeOrtho, further *in-vivo* testing would be required to determine a false-positive rate identification rate for this tool. Because orthologs can function similarly despite low sequence similarity, it is challenging to predict bioengineering outcomes based on protein sequence alone: factors such as the promoter, the transcription start/stop site, copy number, and copy interference can impact physiological outcomes *in vivo*. For this reason, bioengineers having multiple targets identified is helpful to improve the possibility of success. The outputs of AlgaeOrtho provide starting step for the bioengineers to shortlist potential targets with information (including sequence similarity in a heatmap format) to start experimental validation in algae. Indeed, before genetic engineering, researchers could analyze the expression of the putative orthologs via reverse transcription polymerase chain reaction (RT-PCR) to validate their presence and function. In addition, transcriptomics and proteomics studies could provide correlation between the mRNA and protein level, providing strong evidence of functionality between ortholog proteins.

The AlgaeOrtho tool provides simple visualizations to aid bioengineers in the early stages of exploration for protein orthologs of interest, particularly when choosing targets to attain specific genotypes. One feature of the software is that users can easily retrieve the tree file (in the text-based Newick file format) and the percent identity matrix to visualize in another software. A future improvement of the tool could be to have the names automatically standardized to the common names of the species, for easier user readability. Currently, the tool will only display the JGI nomenclature of the species and protein, as listed in the protein sequence (amino acid FASTA) files provided by the user. To convert from these short-hand species and protein names to the scientifically agreed upon species names and protein annotations, the user must search for the abbreviated labels (for example, “ChloA99”) in JGI’s PhycoCosm, and note this name manually. Similarly, the protein codes (for example “41,714”), must be searched manually in the PhycoCosm entry for a particular species. The protein labels are only specific within an algal strain (for example, there may be two different proteins labeled “41,714” in two different algal species). Alternate use-cases for AlgaeOrtho could expand its utility. For example, an evolutionary biologist could use this tool to look at the sequence-similarity of a protein of interest in diverging algae species when doing an experiment on protein divergence. Additionally, it could be useful to use this tool for bacterial, or potentially even plant, and fungal proteomes. Because the goal of bioengineering is to select a gene target, a future feature of this tool would be the ability to look for groups of similar gene sequences and align gene groups that encode for proteins of the same function.

## Data Availability

The original contributions presented in the study are included in the article/Supplementary material, further inquiries can be directed to the corresponding author.

## Author disclaimer

The views expressed in the article do not necessarily represent the views of the DOE or the U.S. Government. The U.S. Government retains and the publisher, by accepting the article for publication, acknowledges that the U.S. Government retains a nonexclusive, paid-up, irrevocable, worldwide license to publish or reproduce the published form of this work, or allow others to do so, for U.S. Government purposes.

## References

[ref1] Altenhoff A. M. Schneider A. Gonnet G. H. Dessimoz C. (2011). OMA 2011: orthology inference among 1000 complete genomes. Nucleic Acids Res. 39, D289–D294. doi: 10.1093/nar/gkq1238, PMID: 21113020 PMC3013747

[ref2] Altschul S. F. Gish W. Miller W. Myers E. W. Lipman D. J. (1990). Basic local alignment search tool. J. Mol. Biol. 215, 403–410. doi: 10.1016/S0022-2836(05)80360-2 2231712

[ref1000] Amaral R. Fawley K. P. Němcová Y. Ševčíková T. Lukešová A. Fawley M. W.. (2020). Towards modern classification of eustigmatophytes, including the description of Neomonodaceae, fam. nov. and three new genera. J Phycol. 56, 630–648. doi: 10.1111/jpy.12980 32068883 PMC7987219

[ref3] Araújo R. Vázquez Calderón F. Sánchez López J. Azevedo I. C. Bruhn A. Fluch S.. (2021). Current status of the algae production industry in Europe: an emerging sector of the blue bioeconomy. Front. Mar. Sci. 7:626389. doi: 10.3389/fmars.2020.626389

[ref4] Arora N. Pienkos P. T. Pruthi V. Poluri K. M. Guarnieri M. T. (2018). Leveraging algal omics to reveal potential targets for augmenting TAG accumulation. Biotechnol. Adv. 36, 1274–1292. doi: 10.1016/j.biotechadv.2018.04.005, PMID: 29678388

[ref5] Arriola M. B. Velmurugan N. Zhang Y. Plunkett M. H. Hondzo H. Barney B. M. (2018). Genome sequences of *Chlorella sorokiniana* UTEX 1602 and *Micractinium conductrix* SAG 241.80: implications to maltose excretion by a green alga. Plant J. 93, 566–586. doi: 10.1111/tpj.13789, PMID: 29178410

[ref6] Banerjee C. Singh P. K. Shukla P. (2016). Microalgal bioengineering for sustainable energy development: recent transgenesis and metabolic engineering strategies. Biotechnol. J. 11, 303–314. doi: 10.1002/biot.201500284, PMID: 26844808

[ref7] Blaby-Haas C. E. Merchant S. S. (2019). Comparative and functional algal genomics. Annu. Rev. Plant Biol. 70, 605–638. doi: 10.1146/annurev-arplant-050718-095841, PMID: 30822111

[ref8] Blanc G. Duncan G. Agarkova I. Borodovsky M. Gurnon J. Kuo A.. (2010). The *Chlorella variabilis* NC64A genome reveals adaptation to Photosymbiosis, coevolution with viruses, and cryptic sex. Plant Cell 22, 2943–2955. doi: 10.1105/tpc.110.076406, PMID: 20852019 PMC2965543

[ref9] Borowitzka M. A. (2018). “Biology of microalgae” in Microalgae in health and disease prevention (London, United Kingdom: Elsevier), 23–72.

[ref10] Chen X. Han H. Jiang P. Nie L. Bao H. Fan P.. (2011). Transformation of β-lycopene cyclase genes from *Salicornia europaea* and Arabidopsis conferred salt tolerance in Arabidopsis and tobacco. Plant Cell Physiol. 52, 909–921. doi: 10.1093/pcp/pcr043, PMID: 21471119

[ref11] Chen J. Li J. Dong W. Zhang X. Tyagi R. D. Drogui P.. (2018). The potential of microalgae in biodiesel production. Renew. Sust. Energ. Rev. 90, 336–346. doi: 10.1016/j.rser.2018.03.073

[ref12] Choi B. Y. Kim H. Shim D. Jang S. Yamaoka Y. Shin S.. (2022). The *Chlamydomonas* bZIP transcription factor BLZ8 confers oxidative stress tolerance by inducing the carbon-concentrating mechanism. Plant Cell 34, 910–926. doi: 10.1093/plcell/koab293, PMID: 34893905 PMC8824676

[ref13] Cock J. M. Sterck L. Rouzé P. Scornet D. Allen A. E. Amoutzias G.. (2010). The Ectocarpus genome and the independent evolution of multicellularity in brown algae. Nature 465, 617–621. doi: 10.1038/nature09016, PMID: 20520714

[ref14] Corteggiani Carpinelli E. Telatin A. Vitulo N. Forcato C. D’Angelo M. Schiavon R.. (2014). Chromosome scale genome assembly and transcriptome profiling of *Nannochloropsis gaditana* in nitrogen depletion. Mol. Plant 7, 323–335. doi: 10.1093/mp/sst120, PMID: 23966634

[ref15] Cosentino S. Iwasaki W. (2019). SonicParanoid: fast, accurate and easy orthology inference. Bioinformatics 35, 149–151. doi: 10.1093/bioinformatics/bty631, PMID: 30032301 PMC6298048

[ref16] Donk E. V. Lürling M. Hessen D. O. Lokhorst G. M. (1997). Altered cell wall morphology in nutrient-deficient phytoplankton and its impact on grazers. Limnol. Oceanogr. 42, 357–364. doi: 10.4319/lo.1997.42.2.0357

[ref17] du Z. Y. Alvaro J. Hyden B. Zienkiewicz K. Benning N. Zienkiewicz A.. (2018). Enhancing oil production and harvest by combining the marine alga *Nannochloropsis oceanica* and the oleaginous fungus *Mortierella elongata*. Biotechnol. Biofuels 11:174. doi: 10.1186/s13068-018-1172-2, PMID: 29977335 PMC6013958

[ref18] Emms D. M. Kelly S. (2015). OrthoFinder: solving fundamental biases in whole genome comparisons dramatically improves orthogroup inference accuracy. Genome Biol. 16:157. doi: 10.1186/s13059-015-0721-2, PMID: 26243257 PMC4531804

[ref19] Emms D. M. Kelly S. (2019). OrthoFinder: phylogenetic orthology inference for comparative genomics. Genome Biol. 20:238. doi: 10.1186/s13059-019-1832-y, PMID: 31727128 PMC6857279

[ref20] Griffiths M. J. Harrison S. T. L. (2009). Lipid productivity as a key characteristic for choosing algal species for biodiesel production. J. Appl. Phycol. 21, 493–507. doi: 10.1007/s10811-008-9392-7

[ref21] Grigoriev I. V. Hayes R. D. Calhoun S. Kamel B. Wang A. Ahrendt S.. (2021). PhycoCosm, a comparative algal genomics resource. Nucleic Acids Res. 49, D1004–D1011. doi: 10.1093/nar/gkaa898, PMID: 33104790 PMC7779022

[ref22] Grigoriev I. V. Nordberg H. Shabalov I. Aerts A. Cantor M. Goodstein D.. (2012). The genome portal of the Department of Energy Joint Genome Institute. Nucleic Acids Res. 40, D26–D32. doi: 10.1093/nar/gkr947, PMID: 22110030 PMC3245080

[ref23] Hamada M. Schröder K. Bathia J. Kürn U. Fraune S. Khalturina M.. (2018). Metabolic co-dependence drives the evolutionarily ancient Hydra–Chlorella symbiosis. eLife 7:e35122. doi: 10.7554/eLife.35122, PMID: 29848439 PMC6019070

[ref24] Jeong S. W. HwangBo K. Lim J. M. Nam S. W. Lee B. S. Jeong B.. (2020). Genetic impairment of cellulose biosynthesis increases Cell Wall fragility and improves lipid extractability from oleaginous alga Nannochloropsis salina. Microorganisms 8:1195. doi: 10.3390/microorganisms8081195, PMID: 32781613 PMC7464416

[ref25] Kim H. S. Park W.-K. Lee B. Seon G. Suh W. I. Moon M.. (2019). Optimization of heterotrophic cultivation of Chlorella sp. HS2 using screening, statistical assessment, and validation. Sci. Rep. 9:19383. doi: 10.1038/s41598-019-55854-9, PMID: 31852948 PMC6920485

[ref26] Kumar G. Shekh A. Jakhu S. Sharma Y. Kapoor R. Sharma T. R. (2020). Bioengineering of microalgae: recent advances, perspectives, and regulatory challenges for industrial application. Front. Bioeng. Biotechnol. 8:914. doi: 10.3389/fbioe.2020.00914, PMID: 33014997 PMC7494788

[ref27] Kwon Y. M. Kim K. W. Choi T.-Y. Kim S. Y. Kim J. Y. H. (2018). Manipulation of the microalgal chloroplast by genetic engineering for biotechnological utilization as a green biofactory. World J. Microbiol. Biotechnol. 34:183. doi: 10.1007/s11274-018-2567-8, PMID: 30478596

[ref28] Lee H. Shin W.-S. Kim Y. U. Jeon S. Kim M. Kang N. K.. (2020). Enhancement of lipid production under heterotrophic conditions by overexpression of an endogenous bZIP transcription factor in *Chlorella* sp. HS2. J. Microbiol. Biotechnol. 30, 1597–1606. doi: 10.4014/jmb.2005.05048, PMID: 32807753 PMC9728203

[ref29] Li L. Stoeckert C. J. Roos D. S. (2003). OrthoMCL: identification of Ortholog groups for eukaryotic genomes. Genome Res. 13, 2178–2189. doi: 10.1101/gr.1224503, PMID: 12952885 PMC403725

[ref30] Liang M. Lu Y. Chen H. Jiang J. (2017). The salt-regulated element in the promoter of lycopene β-cyclase gene confers a salt regulatory pattern in carotenogenesis of *Dunaliella bardawil*. Environ. Microbiol. 19, 982–989. doi: 10.1111/1462-2920.13539, PMID: 27657551

[ref31] Mathimani T. Mallick N. (2018). A comprehensive review on harvesting of microalgae for biodiesel – key challenges and future directions. Renew. Sust. Energ. Rev. 91, 1103–1120. doi: 10.1016/j.rser.2018.04.083

[ref32] Nannochloropsis oceanica CCMP1779 v2.0 (2018). Available at: https://phycocosm.jgi.doe.gov/Nanoce1779_2/Nanoce1779_2.home.html (Accessed December 5, 2024).

[ref33] Nishitsuji K. Arimoto A. Higa Y. Mekaru M. Kawamitsu M. Satoh N.. (2019). Draft genome of the brown alga, Nemacystus decipiens, Onna-1 strain: fusion of genes involved in the sulfated fucan biosynthesis pathway. Sci. Rep. 9:4607. doi: 10.1038/s41598-019-40955-2, PMID: 30872679 PMC6418280

[ref34] Nishitsuji K. Arimoto A. Iwai K. Sudo Y. Hisata K. Fujie M.. (2016). A draft genome of the brown alga, Cladosiphon okamuranus, S-strain: a platform for future studies of 'mozuku' biology. DNA Res. 23, 561–570. doi: 10.1093/dnares/dsw039, PMID: 27501718 PMC5144679

[ref35] Nordberg H. Cantor M. Dusheyko S. Hua S. Poliakov A. Shabalov I.. (2014). The genome portal of the Department of Energy Joint Genome Institute: 2014 updates. Nucl. Acids Res. 42, D26–D31. doi: 10.1093/nar/gkt1069 24225321 PMC3965075

[ref36] Ochromonas sp. CCMP1393 v1.4 (2018). Available at: https://phycocosm.jgi.doe.gov/Ochro1393_1_4/Ochro1393_1_4.home.html (Accessed December 5, 2024).

[ref37] Ohan J. A. Hovde B. T. Zhang X. L. Davenport K. W. Chertkov O. Han C.. (2019). Nuclear genome assembly of the microalga Nannochloropsis salina CCMP1776. Microbiol. Resour. Announc. 8, e00750–e00719. doi: 10.1128/MRA.00750-19, PMID: 31672738 PMC6953503

[ref38] Ostlund G. Schmitt T. Forslund K. Kostler T. Messina D. N. Roopra S.. (2010). InParanoid 7: new algorithms and tools for eukaryotic orthology analysis. Nucleic Acids Res. 38, D196–D203. doi: 10.1093/nar/gkp931, PMID: 19892828 PMC2808972

[ref39] Peterson M. E. Chen F. Saven J. G. Roos D. S. Babbitt P. C. Sali A. (2009). Evolutionary constraints on structural similarity in orthologs and paralogs. Protein Sci. 18, 1306–1315. doi: 10.1002/pro.143, PMID: 19472362 PMC2774440

[ref40] Remm M. Storm C. E. V. Sonnhammer E. L. L. (2001). Automatic clustering of orthologs and in-paralogs from pairwise species comparisons. J. Mol. Biol. 314, 1041–1052. doi: 10.1006/jmbi.2000.5197, PMID: 11743721

[ref41] Ren Y. Sun H. Deng J. Huang J. Chen F. (2021). Carotenoid production from microalgae: biosynthesis, salinity responses and novel biotechnologies. Mar. Drugs 19:713. doi: 10.3390/md19120713, PMID: 34940712 PMC8708220

[ref42] Rost B. (1999). Twilight zone of protein sequence alignments. Protein Eng. Des. Sel. 12, 85–94. doi: 10.1093/protein/12.2.85, PMID: 10195279

[ref43] Schwartz A. S. Brown R. Ajjawi I. McCarren J. Atilla S. Bauman N.. (2018). Complete genome sequence of the model oleaginous alga *Nannochloropsis gaditana* CCMP1894. Genome Announc. 6, e01448–e01417. doi: 10.1128/genomeA.01448-17, PMID: 29449398 PMC5814478

[ref44] Schwede S. Rehman Z.-U. Gerber M. Theiss C. Span R. (2013). Effects of thermal pretreatment on anaerobic digestion of *Nannochloropsis salina* biomass. Bioresour. Technol. 143, 505–511. doi: 10.1016/j.biortech.2013.06.043 23831893

[ref45] Shan T. Yuan J. Su L. Li J. Leng X. Zhang Y.. (2020). First genome of the Brown alga *Undaria pinnatifida*: chromosome-level assembly using PacBio and hi-C technologies. Front. Genet. 11:140. doi: 10.3389/fgene.2020.00140, PMID: 32184805 PMC7058681

[ref46] Sievers F. Wilm A. Dineen D. Gibson T. J. Karplus K. Li W.. (2011). Fast, scalable generation of high-quality protein multiple sequence alignments using Clustal omega. Mol. Syst. Biol. 7:539. doi: 10.1038/msb.2011.75, PMID: 21988835 PMC3261699

[ref47] Tanaka T. Maeda Y. Veluchamy A. Tanaka M. Abida H. Maréchal E.. (2015). Oil accumulation by the oleaginous diatom *Fistulifera solaris* as revealed by the genome and transcriptome. Plant Cell 27, 162–176. doi: 10.1105/tpc.114.135194, PMID: 25634988 PMC4330590

[ref48] The Pandas Development Team (2024). pandas-dev/pandas: Pandas. doi: 10.5281/ZENODO.3509134

[ref49] Verdelho Vieira V. Cadoret J.-P. Acien F. G. Benemann J. (2022). Clarification of Most relevant concepts related to the microalgae production sector. PRO 10:175. doi: 10.3390/pr10010175

[ref50] Wichuk K. Brynjólfsson S. Fu W. (2014). Biotechnological production of value-added carotenoids from microalgae: emerging technology and prospects. Bioengineered 5, 204–208. doi: 10.4161/bioe.28720, PMID: 24691165 PMC4101014

[ref51] Zhao Z. Liu Z. Mao X. (2020). Biotechnological advances in lycopene β-Cyclases. J. Agric. Food Chem. 68, 11895–11907. doi: 10.1021/acs.jafc.0c04814, PMID: 33073992

[ref52] Zhu M. Meng X. Cai J. Li G. Dong T. Li Z. (2018). Basic leucine zipper transcription factor SlbZIP1 mediates salt and drought stress tolerance in tomato. BMC Plant Biol. 18:83. doi: 10.1186/s12870-018-1299-0, PMID: 29739325 PMC5941487

