# Supplementary material for: AlgaeOrtho, a bioinformatics tool for processing ortholog inference results in algae
Source: Front Microbiol. 2025 Mar 4;16:1541898. doi: 10.3389/fmicb.2025.1541898 (PMC11913701; doi:10.3389/fmicb.2025.1541898)
Supplement: Supplementary file 1 [file Data_Sheet_1.docx]

**Supplementary Materials**

**Section S1:** How to download sequences in batch from JGI’s PhycoCosm resource?

To download sequences in batch from JGI’s PhycoCosm resource use the following link: <https://phycocosm.jgi.doe.gov/phycocosm/home> (Supplementary Figure S1)

From the main page, a clade can be selected, and from there a dropdown menu will appear with the option to “Search” (Supplementary Figure S2-S3). Select “Search” to look for a specific group of annotated proteins of interest (Supplementary Figure S4). A protein FASTA can be downloaded from this page, to be used as a “query” file in the AlgaeOrtho application.

**Supplementary Figure S1:** A screenshot of PhycoCosm as of Dec 13 2023.


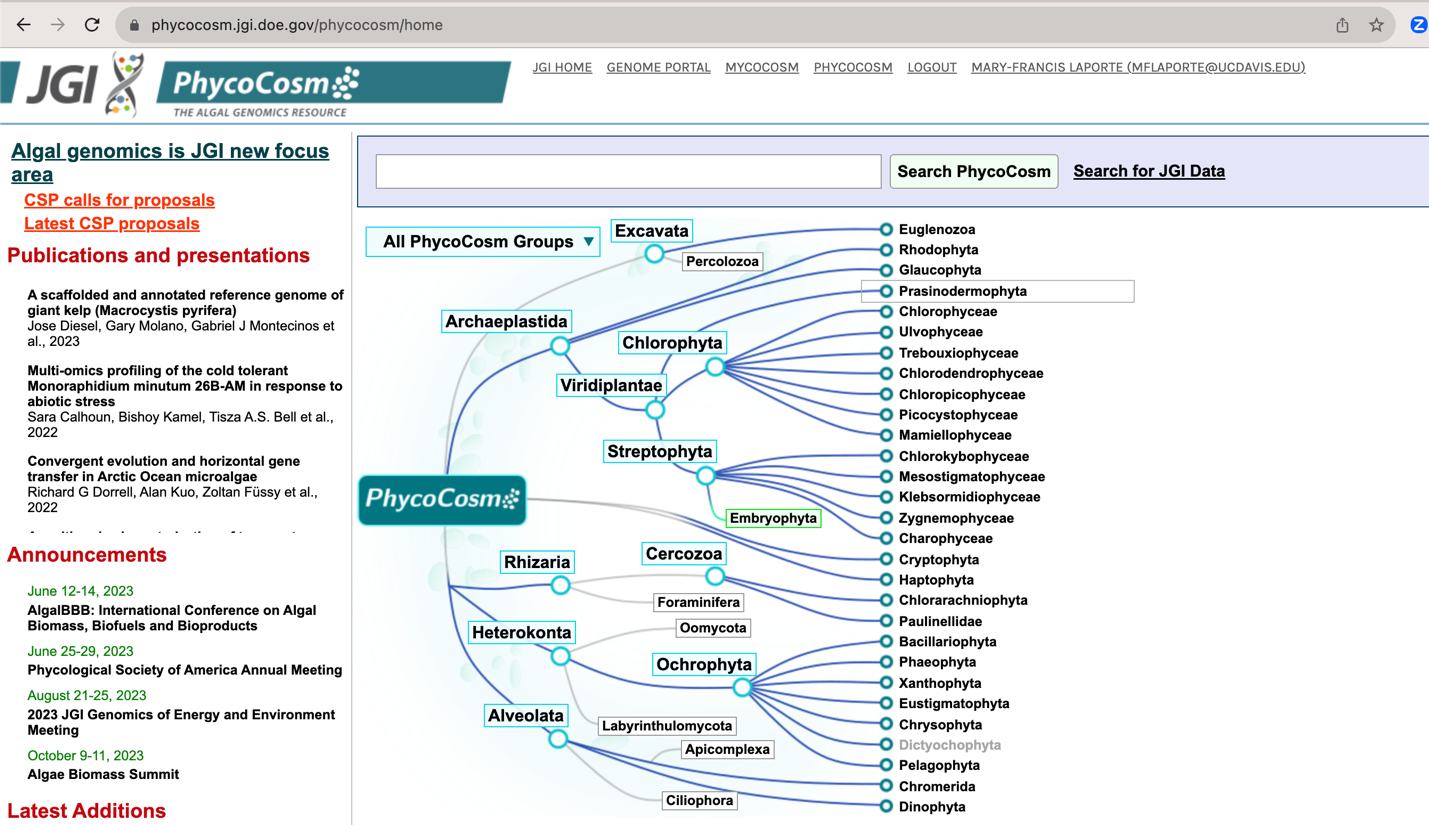


**Supplementary Figure S2:** The dropdown menu for Chlorophyta, from JGI’s PhycoCosm as of Dec 13 2023.


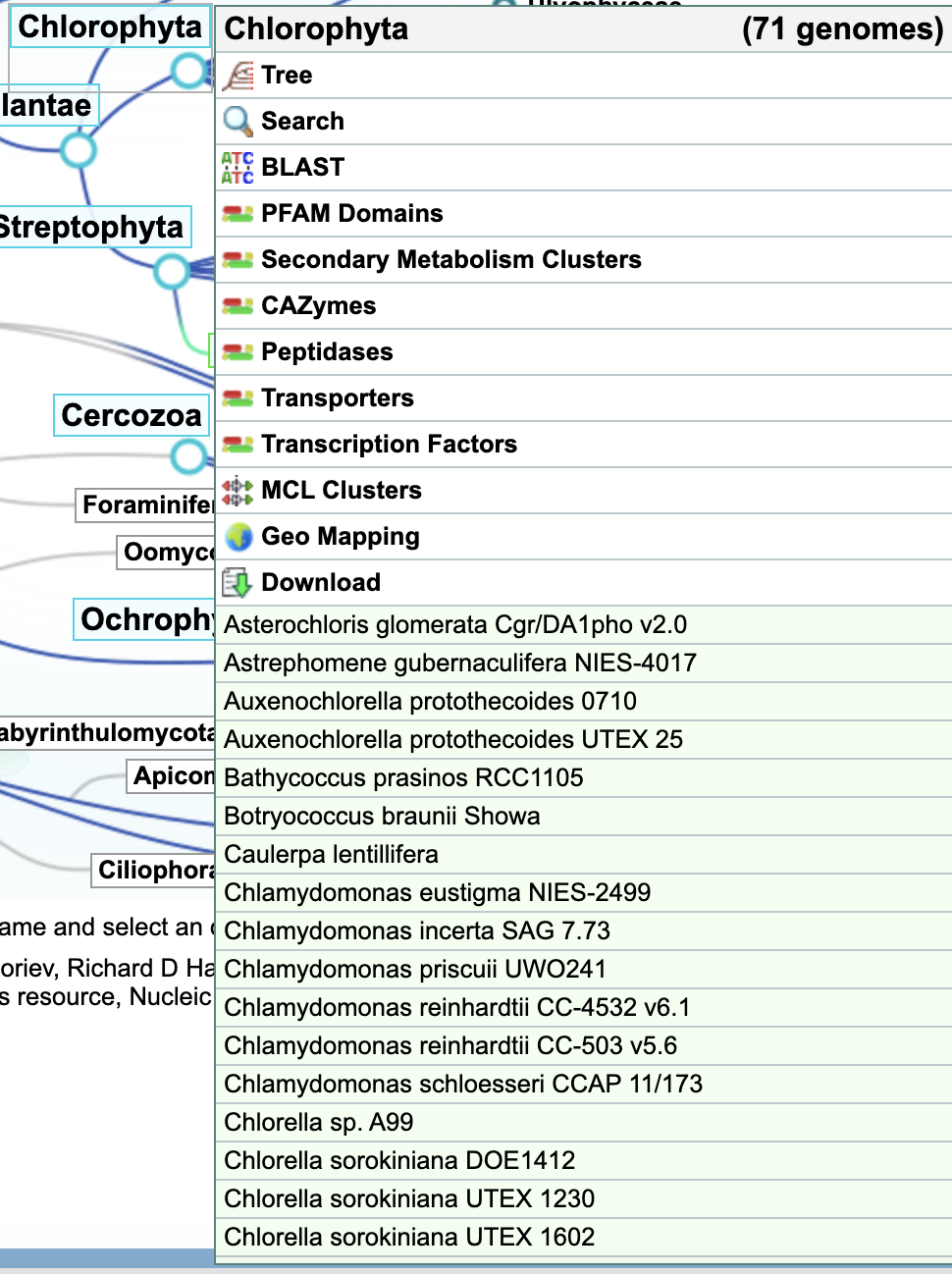


**Supplementary Figure S3:** A search query for bZIP in Chlorophyta, as well as the dark-gray dropdown menu to select and download protein sequences of interest as a protein FASTA file, from JGI’s PhycoCosm as of Dec 13 2023.


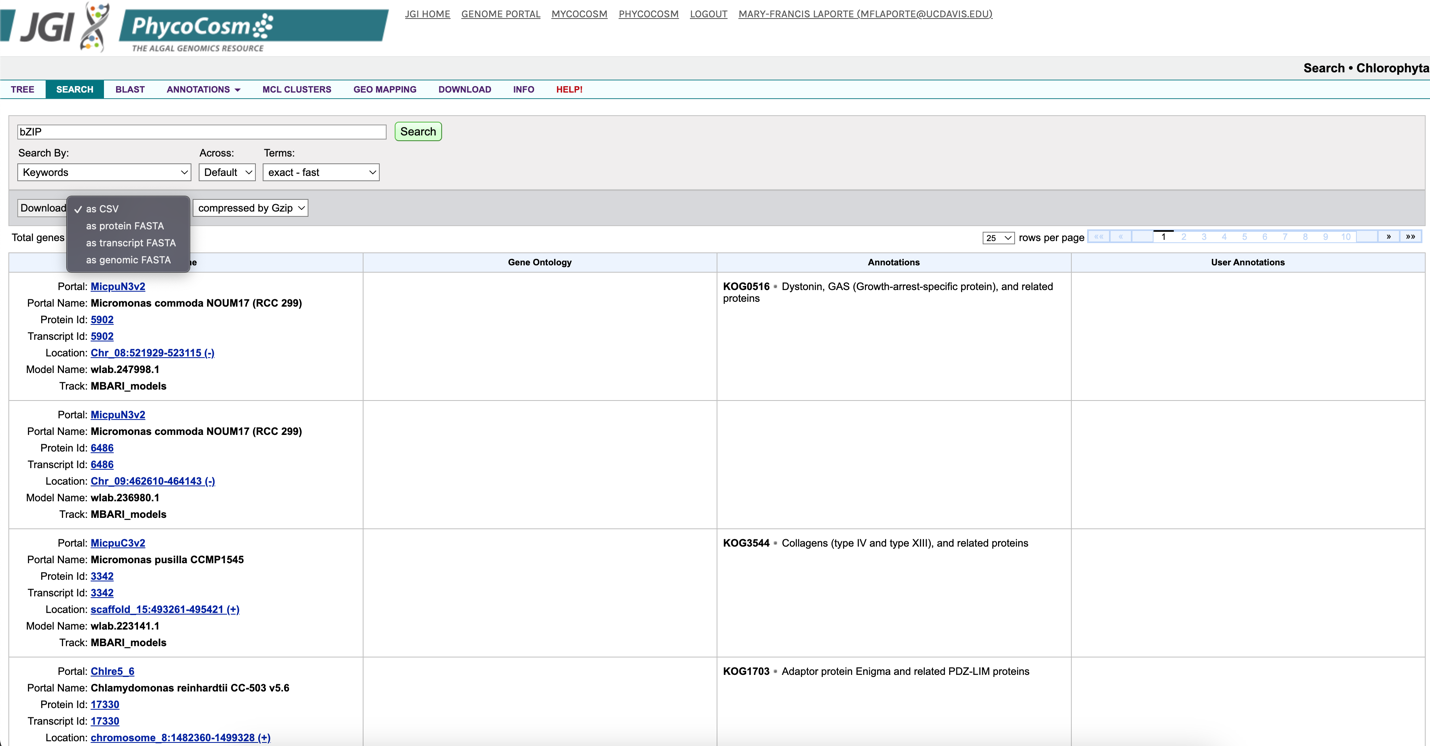


**Section S2:** How to generate ortholog groups using SonicParanoid?

The application works with the ortholog group we generated using the sequences in Supplementary Table S1 using SonicParanoid. If a different ortholog group is desired, the following are instructions to generate that file. Instructions for download and installation of Sonic Paranoid are found on the website: <http://iwasakilab.k.u-tokyo.ac.jp/sonicparanoid/>

The input file needed to run SonicParanoid is a folder that contains only the relevant aa.fasta protein sequences from JGI (Supplementary Figure S3). The output generated from SonicParanoid constitutes a folder containing a series of files, including one called “ortholog_groups.csv” to be used with the AlgaeOrtho application if desired (Supplementary Figure S4).

The following line of code should be run in the command line, and it identifies orthologous groups for all proteins included in the input folder using SonicParanoid for as many algal species as desired.

$ sonicparanoid -i InputDirectory -o OutputDirectoryName -p NAME --output-pairs

Note: this line of BASH code (to be run in the terminal) requires that SonicParanoid is already installed.

InputDirectory denotes the path to the folder that contains only the relevant aa.fasta protein sequences from JGI

OutputDirectoryName is the path to the output folder (this must be a path to a directory that already exists.

NAME is the name you'd like to give to this analysis for your internal records.

**Supplementary Table S1:**

List of the 92 Algae Protein Files used with SonicParanoid to be the basis of analysis for the query search. All files were accessed using JGI’s Phycocosm portal and are searchable using the name (the set of characters before the first underscore “_”).

| Astpho2_GeneCatalog_proteins_20111209.aa.fasta |
| --- |
| Auran1_GeneCatalog_proteins.FilteredModels3.fasta |
| Auxeprot1_GeneCatalog_proteins_20170909.aa.fasta |
| Batpra1_GeneCatalog_proteins_20180426.aa.fasta |
| Botrbrau1_GeneCatalog_proteins_20200805.aa.fasta |
| Caulen1_GeneCatalog_proteins_20200307.aa.fasta |
| Chabra1_GeneCatalog_proteins_20200807.aa.fasta |
| Chlat1_GeneCatalog_proteins_20200803.aa.fasta |
| Chleu1_GeneCatalog_proteins_20200804.aa.fasta |
| Chlin1_GeneCatalog_proteins_20200804.aa.fasta |
| ChlNC64A_1_GeneCatalog_Chlorella_NC64A.best_proteins.fasta |
| ChloA99_1_GeneCatalog_proteins_20200807.aa.fasta |
| ChloDOE1412_1_GeneCatalog_proteins_20200805.aa.fasta |
| Chloso1228_1_GeneCatalog_proteins_20200805.aa.fasta |
| Chloso1230_1_GeneCatalog_proteins_20200805.aa.fasta |
| Chloso1602_1_GeneCatalog_proteins_20200805.aa.fasta |
| Chlpri1_GeneCatalog_proteins_20200307.aa.fasta |
| Chlre5_6_GeneCatalog_proteins_20200117.aa.fasta |
| Chlsc1_GeneCatalog_proteins_20200804.aa.fasta |
| ChlUWO241_1_GeneCatalog_proteins_20220210.aa.fasta |
| Chrzof1_GeneCatalog_proteins_20190731.aa.fasta |
| Claok1_GeneCatalog_proteins_20200804.aa.fasta |
| Coc_C169_1_GeneCatalog_Coccomyxa_C169_v2_filtered_proteins.fasta |
| Cyapar1_GeneCatalog_proteins_20200807.aa.fasta |
| Cyccr1_GeneCatalog_proteins_20200805.aa.fasta |
| Ectsil1_GeneCatalog_proteins_20171109.aa.fasta |
| Edade1_GeneCatalog_proteins_20200805.aa.fasta |
| Fisso1_GeneCatalog_proteins_20200805.aa.fasta |
| Gonpec1_GeneCatalog_proteins_20180501.aa.fasta |
| Klenit1_GeneCatalog_proteins_20200807.aa.fasta |
| Mesen1_GeneCatalog_proteins_20200803.aa.fasta |
| Mesovir1_GeneCatalog_proteins_20200803.aa.fasta |
| Mesvir1_GeneCatalog_proteins_20200803.aa.fasta |
| Micco1_GeneCatalog_proteins_20200804.aa.fasta |
| MicpuC3v2_GeneCatalog_proteins_20160125.aa.fasta |
| MicpuN3v2_GeneCatalog_proteins_20160404.aa.fasta |
| MicrAD1_1_GeneCatalog_proteins_20220220.aa.fasta |
| MicrYARC1_GeneCatalog_proteins_20220803.aa.fasta |
| Monneg1_GeneCatalog_proteins_20170920.aa.fasta |
| Nangad1_GeneCatalog_proteins_20180307.aa.fasta |
| Nansal1776_1_GeneCatalog_proteins_20200807.aa.fasta |
| Nemde1_GeneCatalog_proteins_20200804.aa.fasta |
| Nithil2_GeneCatalog_proteins_20201125.aa.fasta |
| Ochro2298_1_GeneCatalog_proteins_20160817.aa.fasta |
| Ost9901_3_GeneCatalog_O.lucimarinus.FM.aa.fasta |
| Ostta1115_2_GeneCatalog_proteins_20170131.aa.fasta |
| Ostta4221_3_GeneCatalog_proteins_20161028.aa.fasta |
| Pelago2097_1_GeneCatalog_proteins_20160408.aa.fasta |
| Phypa1_1_GeneCatalog_proteins.1.FilteredModels.fasta |
| Pico_ML_1_GeneCatalog_proteins_20200807.aa.fasta |
| Picre1_GeneCatalog_proteins_20200805.aa.fasta |
| Picsp_1_GeneCatalog_proteins_20170909.aa.fasta |
| Rapsub1_GeneCatalog_proteins_20191119.aa.fasta |
| Sacja1_GeneCatalog_proteins_20210418.aa.fasta |
| Scesp_1_GeneCatalog_proteins_20191220.aa.fasta |
| Semro1_GeneCatalog_proteins_20200811.aa.fasta |
| Spimu1_GeneCatalog_proteins_20200803.aa.fasta |
| Tetso1_GeneCatalog_proteins_20200805.aa.fasta |
| Tetstr1_GeneCatalog_proteins_20200807.aa.fasta |
| Thaoce1_GeneCatalog_proteins_20171026.aa.fasta |
| TrebA12_1_GeneCatalog_proteins_20200804.aa.fasta |
| Trimin1_GeneCatalog_proteins_20201122.aa.fasta |
| Ulvmu1_GeneCatalog_proteins_20200803.aa.fasta |
| Undpi1_GeneCatalog_proteins_20200805.aa.fasta |
| Volca2_1_GeneCatalog_proteins_20200117.aa.fasta |
| Dunsal1_1_GeneCatalog_proteins_20200511.aa.fasta |
| Auxpr25_1_GeneCatalog_proteins_20200805.aa.fasta |
| Mintr2_GeneCatalog_proteins_20160618.aa.fasta |
| Ochro1393_1_4_GeneCatalog_proteins_20181204.aa.fasta |
| Parimp1_4_GeneCatalog_proteins_20180208.aa.fasta |
| Sceobl1_GeneCatalog_proteins_20200506.aa.fasta |
| Sceobl1450_1_GeneCatalog_proteins_20200927.aa.fasta |
| Sceobl2630_1_GeneCatalog_proteins_20200906.aa.fasta |
| SceoblDOE13_1_GeneCatalog_proteins_20200927.aa.fasta |
| SceoblEN4_1_GeneCatalog_proteins_20190921.aa.fasta |
| Sobl393_1_GeneCatalog_proteins_20200120.aa.fasta |
| Dicre1_GeneCatalog_proteins_20171101.aa.fasta |
| Nanoce1779_2_GeneCatalog_proteins_20180119.aa.fasta |
| OstRCC809_2_GeneCatalog_OstreococcusRCC809v2.FilteredModels3.proteins.fasta |
| Psemu1_GeneCatalog_proteins_20111011.aa.fasta |
| SymretAf1_GeneCatalog_proteins_20180322.aa.fasta |
| SymretSc1_GeneCatalog_proteins_20180314.aa.fasta |
| SymretSp1_GeneCatalog_proteins_20171013.aa.fasta |
| SymretSw1_GeneCatalog_proteins_20180321.aa.fasta |
| DesarB2533_2_GeneCatalog_proteins_20210517.aa.fasta |
| Enacos1_1_GeneCatalog_proteins_20210913.aa.fasta |
| Flerot1_1_GeneCatalog_proteins_20210723.aa.fasta |
| Meskra657_3_GeneCatalog_proteins_20220510.aa.fasta |
| Monmin1_GeneCatalog_proteins_20190530.aa.fasta |
| Sceobl393_2_GeneCatalog_proteins_20200804.aa.fasta |
| TetrdesSNI2_1_GeneCatalog_proteins_20210729.aa.fasta |
| Tetrobl72_1_GeneCatalog_proteins_20210717.aa.fasta |

**Supplementary Figure S4:** Screenshot of the application: AlgaeOrtho allows for three different settings, the first two case studies, as well as the user’s own protein files of interest


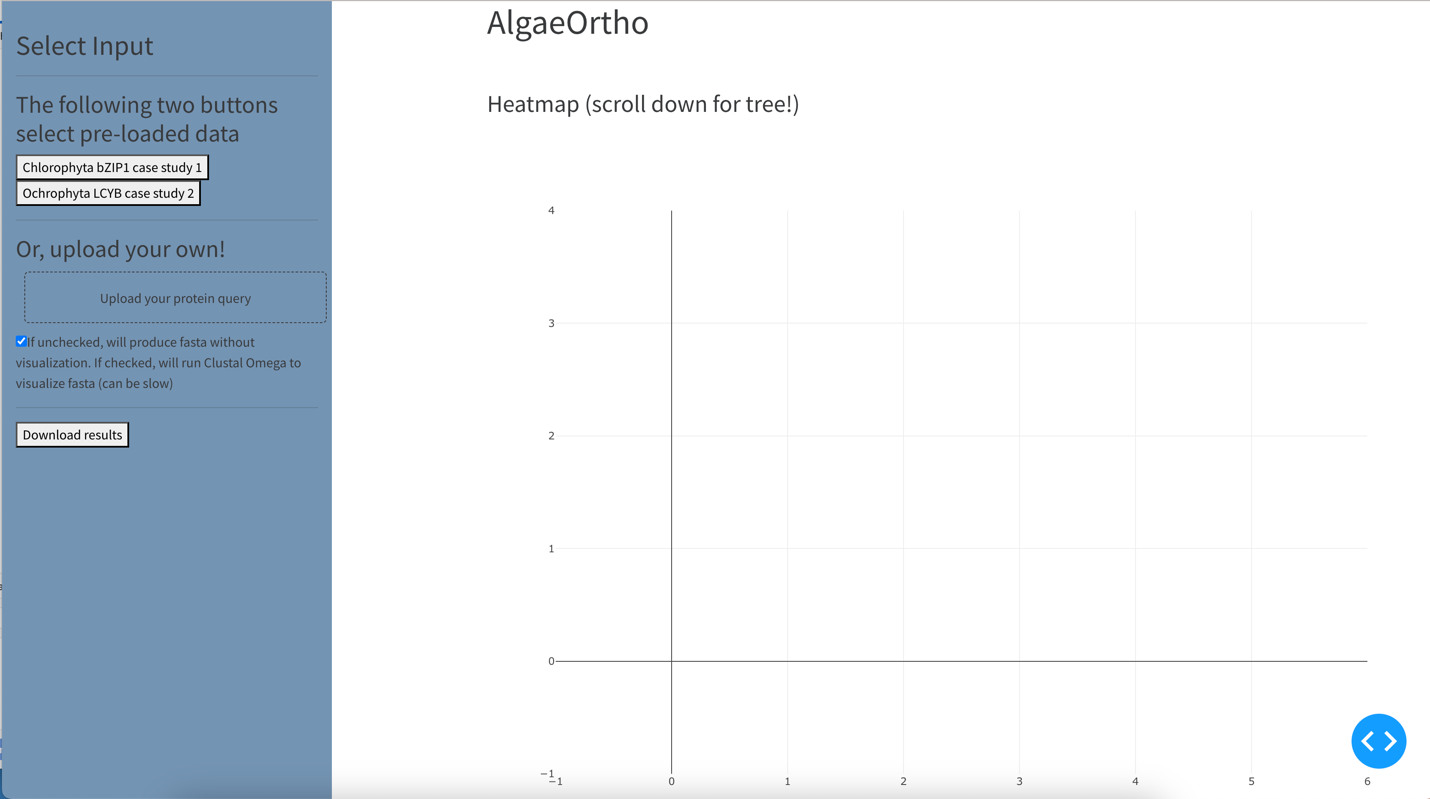


**Section S3:** How to install and use the AlgaeOrtho Application?

**3.1: Helpful software and pre-requisites**

1. Docker: a tool to handle the software prerequisites for the AlgaeOrtho application: <https://hub.docker.com/>. Once Docker is installed, it will handle the Python software installation that our application uses to run. Although it will require the user to run a couple of lines of code in the terminal, this is easier than the user needing to handle software installation and versioning themselves.
2. GitHub: a tool to share code and make it accessible over the internet. The user will want a GitHub account <https://github.com/> to retrieve the code for this application: <https://github.com/laporpe/AlgaeOrtho> . This code can then be downloaded to the user’s device so they can run the code.
3. One way to download the code for the application is through the GitHub Desktop application: <https://desktop.github.com/> . The GitHub Desktop application can be connected with the users’ GitHub account, so that the user can run AlgaeOrtho code (or any other code accessed from GitHub) on their computer. Here are instructions on how to do that: <https://docs.github.com/en/desktop/adding-and-cloning-repositories/cloning-a-repository-from-github-to-github-desktop>
4. Visual Studio Code (VS Code): a code editor that allows the user to open up the AlgaeOrtho files and run the application. VS Code is advantageous because it has a built-in terminal. Once the code has been obtained from Github, the user should open it in VS Code.

**3.2: How to use Docker?**

1. Download Docker <https://hub.docker.com/>
2. Pull the code for the application from Github <https://github.com/laporpe/AlgaeOrtho>.
   1. One way to do this is through using the GitHub Desktop App: <https://desktop.github.com/>. See link in Part 3 above.

1. Open the directory that contains the code for the application locally in VS Code. This can be done by pressing the “Open in Visual Studio Code” button in the GitHub Desktop application (center section, two boxes beneath the “No local changes” heading).
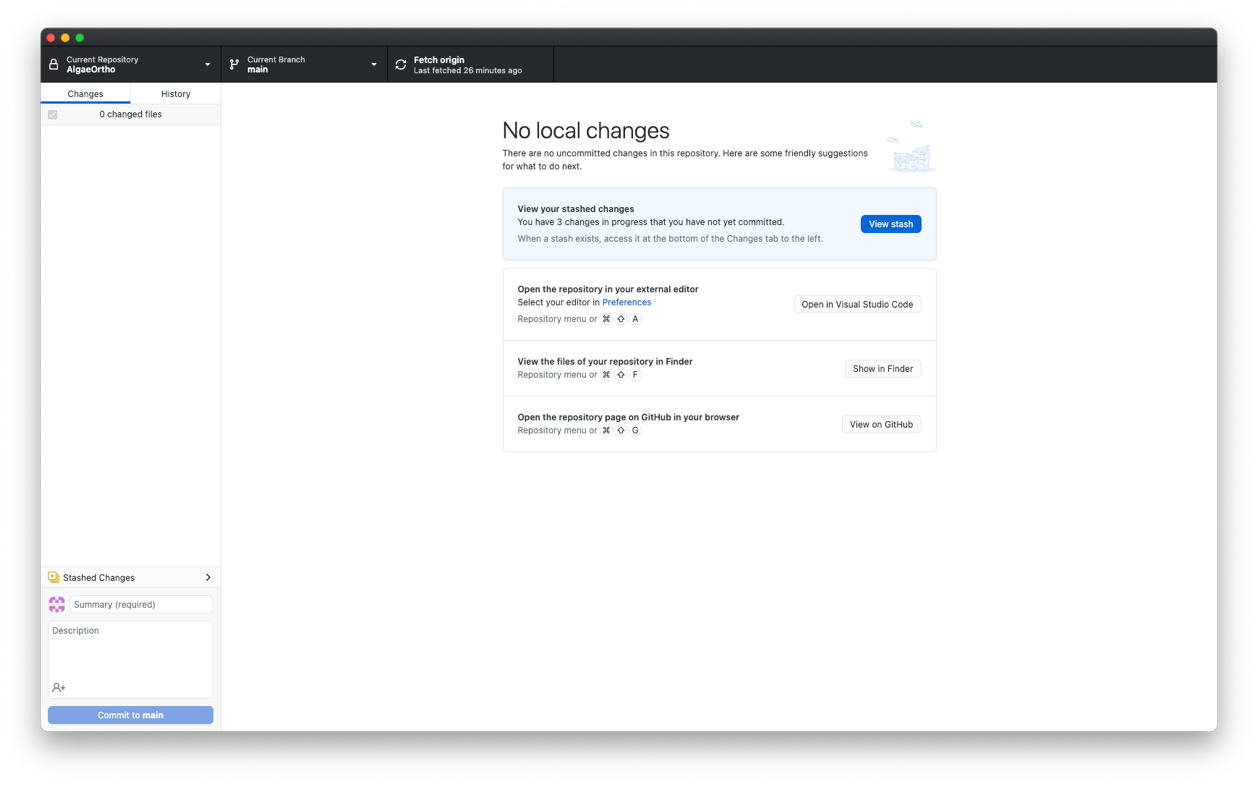

2. Once the application files have been opened in VS Code, navigate to the Terminal in VS code (usually at the bottom of the screen), and run this:

docker compose build

- 1. This command builds the docker container so that all of the necessary prerequisites for this application are pre-installed.


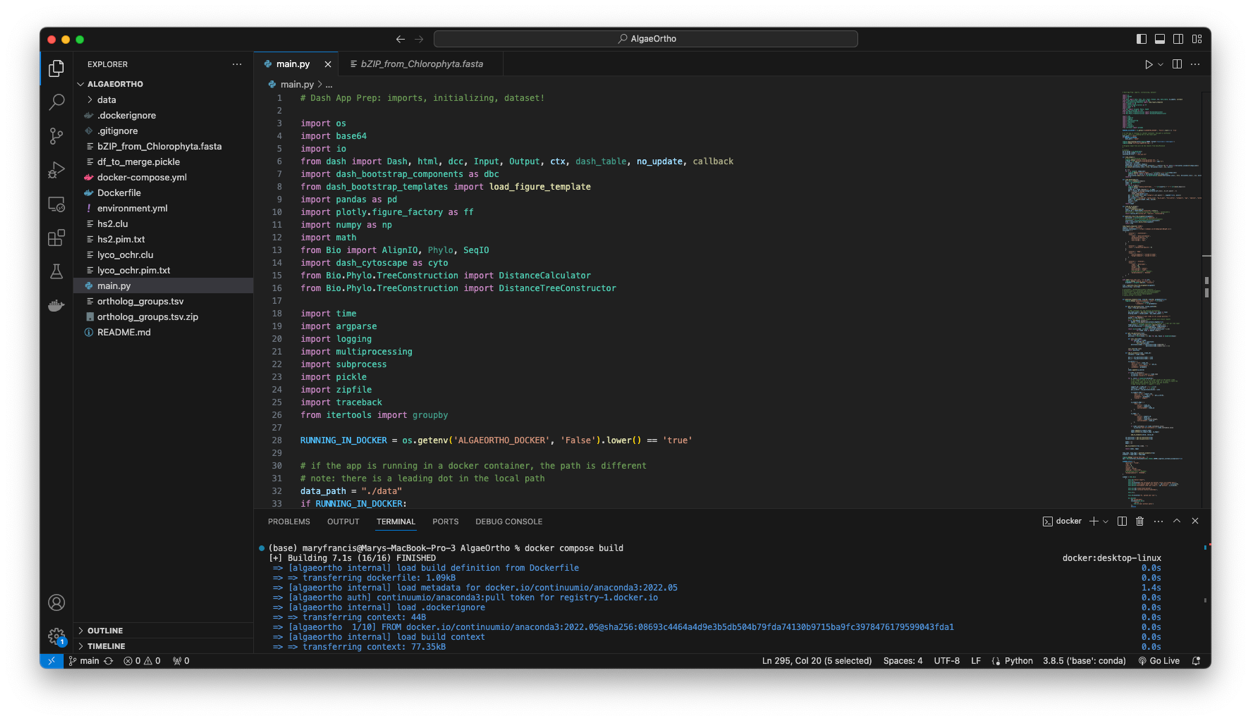


1. Once that command has finished, In the Terminal in VS code (with the application directory open), run this command:

docker compose up


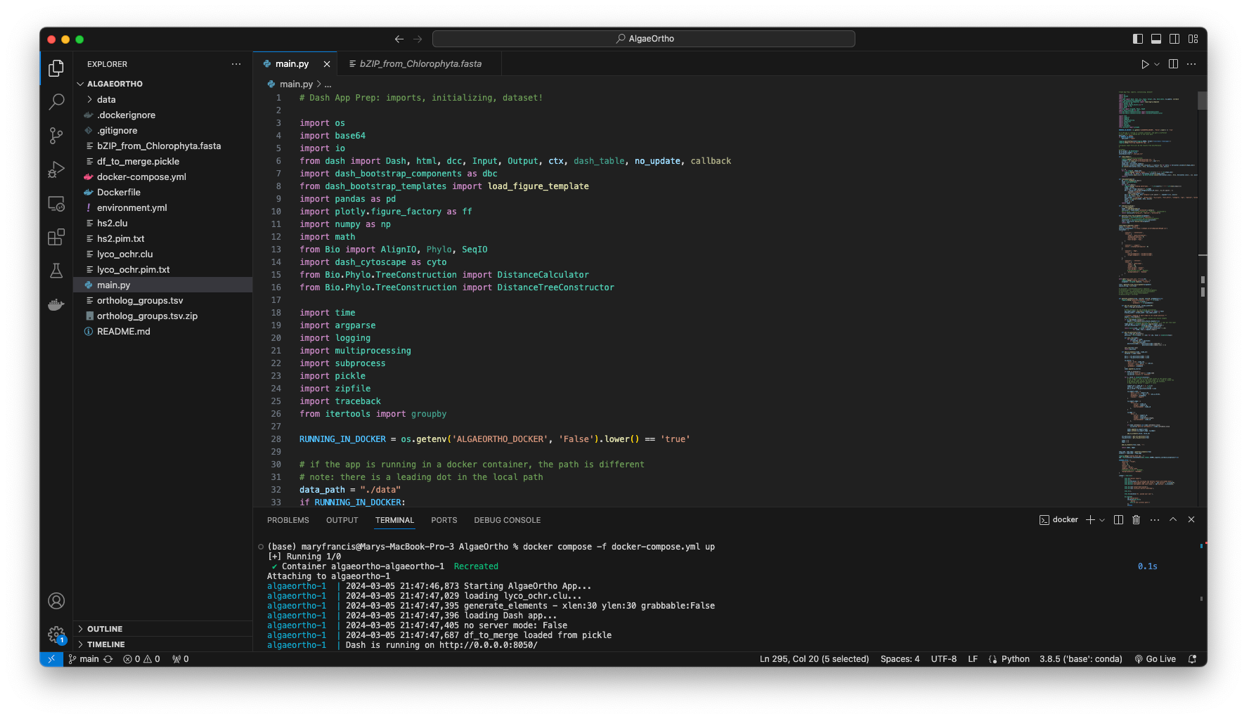


1. This will make the application accessible locally at <http://127.0.0.1:8050> (another equivalent option: localhost:8050)
   1. In other words, type [127.0.0.1:8050](http://127.0.0.1:8050/) (or localhost:8050) into your browser (for example, Google Chrome) and the application will open. The application is running locally, and using the graphical tools from your browser to display the application. As the application is running locally, anything you upload only is being processed on your computer and is not being uploaded online.
2. From here you should be able to use the example buttons as well as upload your own sequences!

**3.3: How to use the Algae Ortho application?**


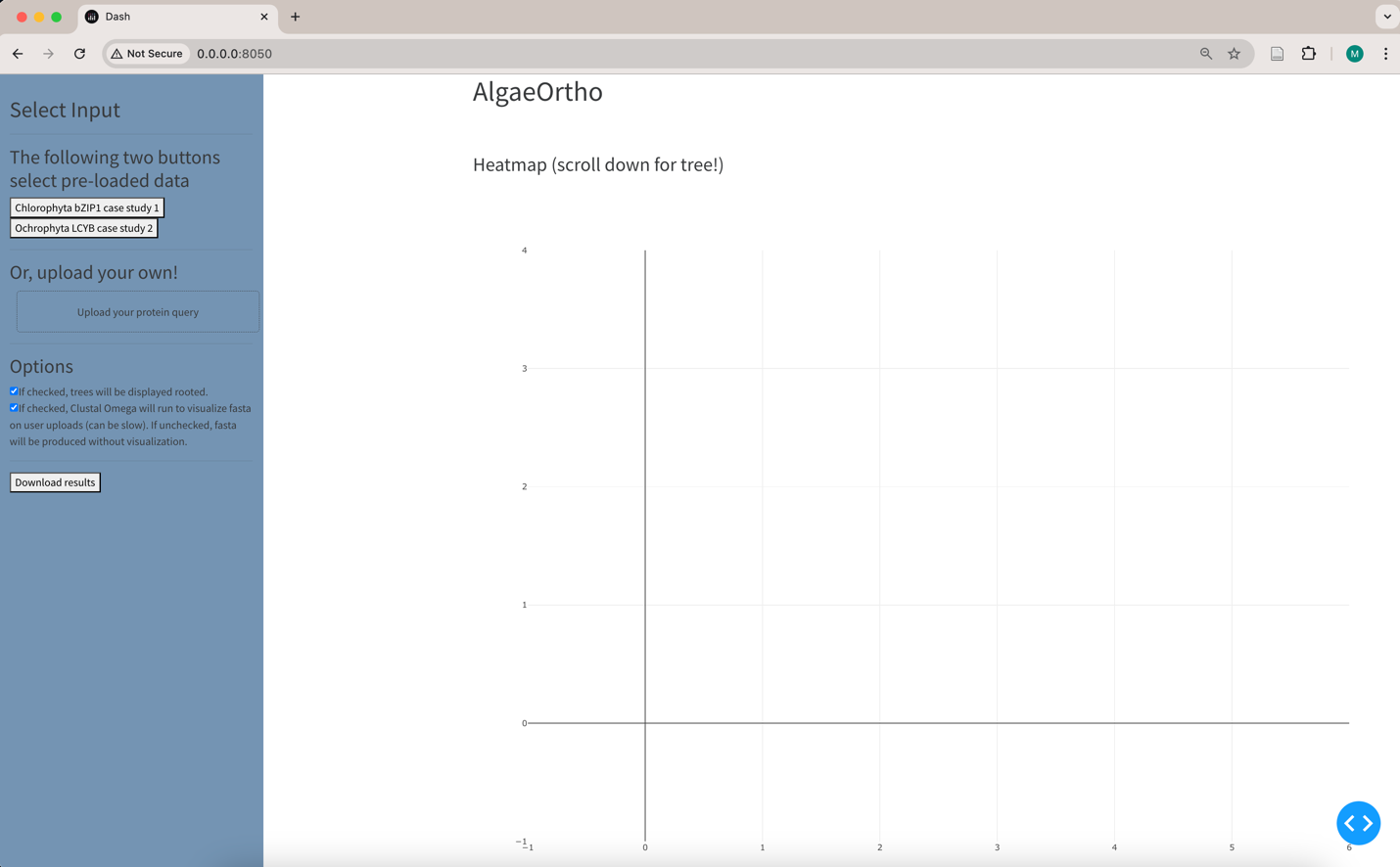


This is how the application will appear when it is first loaded. Select the buttons on the upper left to visualize pre-loaded data (described in the manuscript).

To upload your own data, use the “upload your protein query” button. Upload any protein .fasta file, that does not end each protein with asterisks (*). Try a short file with only a few proteins first, as these run quickly.

Leave the checkbox “unchecked” for the “quick” version of the application, which only finds the putative orthologs from the sonic paranoid results, but does not visualize them. This will allow you to download the resulting protein fasta file using the “download results” button. Leave the checkbox “checked” to visualize the results of the putative ortholog file in the application.

Download all results files by clicking the “download results” button. This will download a folder that needs to be unzipped, that will contain the.fasta file of all putative orthologs, as well as the generated Newick file to visualize a tree of those sequences in your tree visualizer of choice.

Remember to scroll down (on the white-background section of the application) to see the ortholog tree!

The figures can be zoomed in and out using the Dash app controls
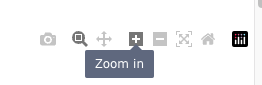


**3.4: Troubleshooting:**

If you are running this on an M1 Mac: the visualization aspect of the application uses a function that cannot work with an M1 Mac.

There is a separate container distributed for users on an M1 Mac, that has a “checkbox unchecked” version of the application. To access this container, follow the instructions above, but replace the docker commands with the following:

docker compose -f docker-compose-noclustalo.yml build

docker compose -f docker-compose-noclustalo.yml up

This specifies the “noclustalo” (non-visualized) version, which should work on all machines.

**Supplementary Table S2:** CesA sequences from the Eustigmatophyta identified via JGI’s PhycoCosm. These proteins sequences were used as input queries to AlgaeOrtho to search for putative orthologs within the 92 studied species mentioned in Supplementary S1.

| FASTA Header | Species | Protein ID | Model Name |
| --- | --- | --- | --- |
| >jgi | NanspC018_1 | 282308 | fgenesh1_kg.4__121__STRG.937.1 |
| >jgi | Nangad1894_1 | 4741 | CM00_9061_g47440.t1 |
| >jgi | Nanoce84910_1 | 6804 | g3710.t1 |
| >jgi | Nansal1776_1_1 | 1798 | NSK_006913.t1 |
| >jgi | VisC74_1 | 15202 | Vischeria_C74_contig_5_g39230.t1 |
| >jgi | Nanoce84910_1 | 5806 | g5962.t1 |
| >jgi | Nangad1 | 9901 | rna3686 |
| >jgi | NanspC018_1 | 289025 | fgenesh1_kg.26__40__STRG.1183.1 |
| >jgi | MonC73_1 | 5240 | Monodopsis_C73_contig_18_g113260.t1 |
| >jgi | MonC73_1 | 10505 | Monodopsis_C73_contig_4_g34350.t1 |
| >jgi | MonC73_1 | 13488 | Monodopsis_C73_contig_9_g65120.t1 |
| >jgi | MonC141_1 | 4838 | Monodopsis_C141_contig_18_g104150.t1 |
| >jgi | MonC141_1 | 8293 | Monodopsis_C141_contig_27_g132460.t1 |
| >jgi | MonC141_1 | 12207 | Monodopsis_C141_contig_6_g44300.t1 |
| >jgi | VisC74_1 | 15708 | Vischeria_C74_contig_6_g45230.t1 |
| >jgi | VisC74_1 | 17264 | Vischeria_C74_contig_8_g62830.t1 |
| >jgi | VisC74_1 | 9412 | Vischeria_C74_contig_24_g164420.t1 |
| >jgi | Nanoce1779_2 | 337008 | CE337007_65371 |
| >jgi | Nangad1894_1 | 3503 | CM00_9058_g35050.t1 |
| >jgi | Nangad1894_1 | 8613 | CM00_9075_g86300.t1 |
| >jgi | MonC73_1 | 13773 | Monodopsis_C73_contig_9_g68070.t1 |
| >jgi | MonC141_1 | 11901 | Monodopsis_C141_contig_6_g41150.t1 |
| >jgi | MonC73_1 | 959 | Monodopsis_C73_contig_1_g09920.t1 |
| >jgi | Nanoce1779_2 | 591253 | fgenesh1_kg.12__470__TRINITY_DN14137_c0_g1_i1 |
| >jgi | Nangad1 | 1618 | rna5640 |
| >jgi | Nangad1894_1 | 3724 | CM00_9058_g37260.t1 |


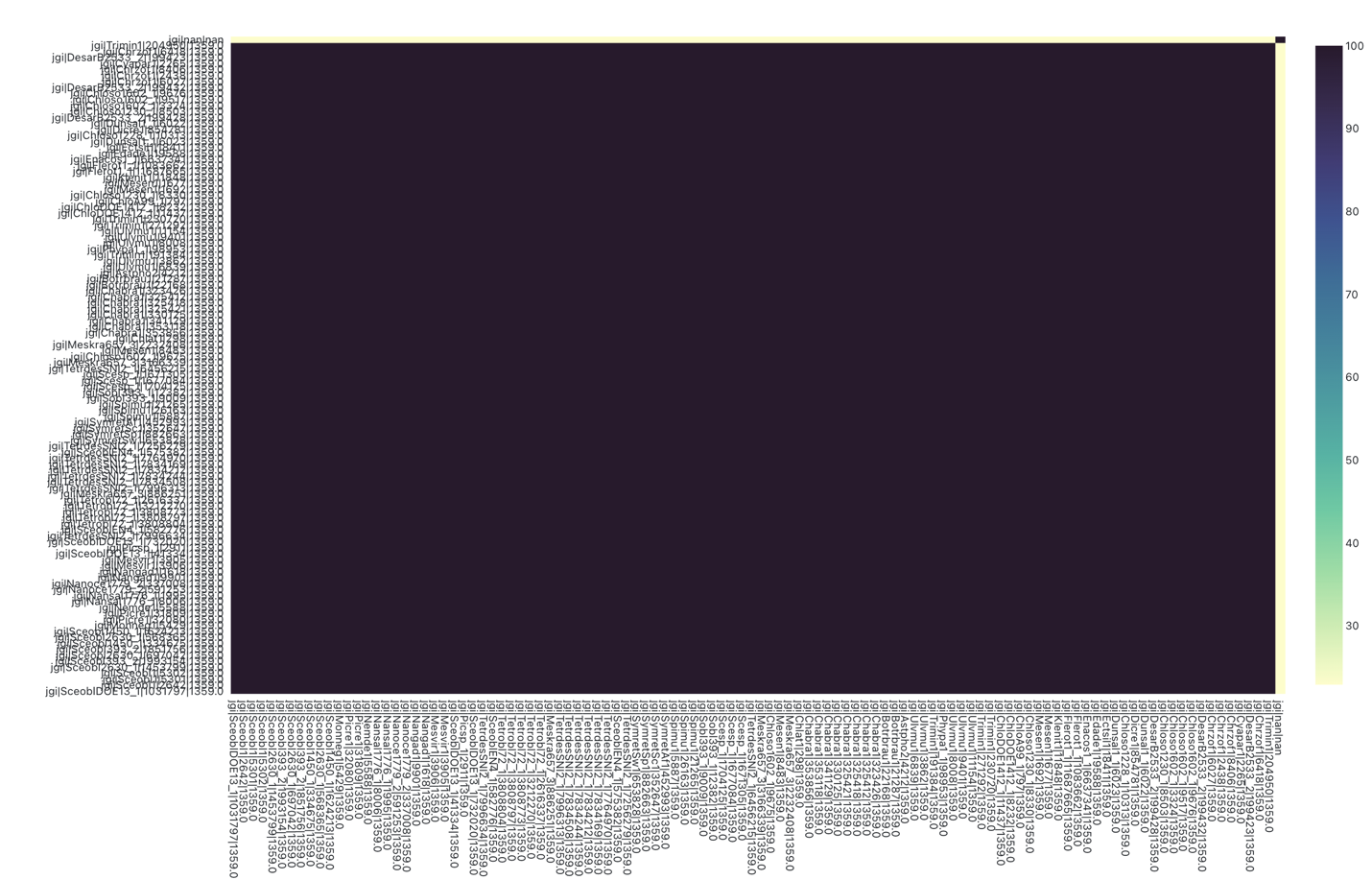


**Supplementary Figure S5:** Heatmap of proteins that are putative orthologs of cellulose synthase proteins from the Eustigmatophyta. The names on each axis reflect the species from which the ortholog sequence was identified. The naming convention of the labels reflects the JGI naming convention of proteins from proteome sequences: <jgi> | <species identification code>| <ortholog group number>| <protein identification code>. This signifies: The origin in the JGI database | a code specific to the JGI system | ortholog number generated by SonicParanoid | a protein code specified by JGI.


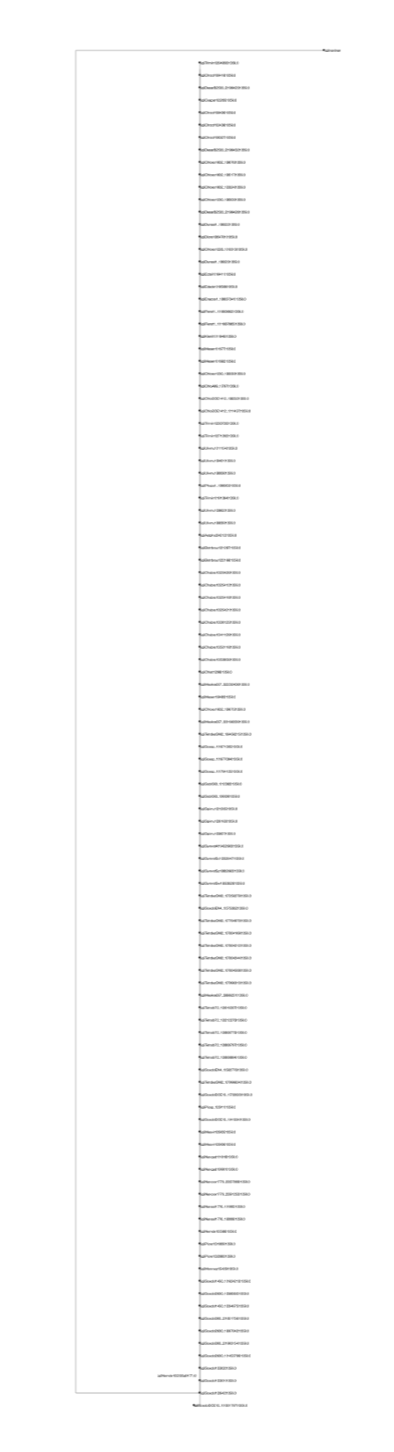


**Supplementary Figure S6:**

Clustering of proteins that are putative orthologs of cellulose synthase proteins from the Eustigmatophyta. The clustering was calculated by Clustal Omega, and the distance calculated by BioPython’s Phylo Tree Construction tools. All entries are equidistant because the sequences have 100% sequence similarity.The names on each axis reflect the species from which the ortholog sequence was identified. The naming convention of the labels reflects the JGI naming convention of proteins from proteome sequences: <jgi>, which denotes a sequence origin of JGI | <species identification code>, originating from the JGI system | <protein identification number>, which is species and JGI specific| <ortholog group number>, which was generated by SonicParanoid.
